# In vivo non-invasive monitoring of dystrophin correction in a new Duchenne muscular dystrophy reporter mouse

**DOI:** 10.1038/s41467-019-12335-x

**Published:** 2019-10-04

**Authors:** Leonela Amoasii, Hui Li, Yu Zhang, Yi-Li Min, Efrain Sanchez-Ortiz, John M. Shelton, Chengzu Long, Alex A. Mireault, Samadrita Bhattacharyya, John R. McAnally, Rhonda Bassel-Duby, Eric N. Olson

**Affiliations:** 1 Department of Molecular Biology, Hamon Center for Regenerative Science and Medicine, Sen. Paul D. Wellstone Muscular Dystrophy Cooperative Research Center, Watertown, MA 02472 USA; 2 Exonics Therapeutics, 490 Arsenal Way, Watertown, MA 02472 USA; 3 Department of Internal Medicine, University of Texas Southwestern Medical Center, 5323 Harry Hines Boulevard, Dallas, TX 75390 USA; 4 Present Address: Leon H. Charney Division of Cardiology, New York University School of Medicine, New York, NY 10016 USA

**Keywords:** Bioluminescence imaging, CRISPR-Cas9 genome editing, Neuromuscular disease

## Abstract

Duchenne muscular dystrophy (DMD) is a fatal genetic disorder caused by mutations in the dystrophin gene. To enable the non-invasive analysis of DMD gene correction strategies in vivo, we introduced a luciferase reporter in-frame with the C-terminus of the dystrophin gene in mice. Expression of this reporter mimics endogenous dystrophin expression and DMD mutations that disrupt the dystrophin open reading frame extinguish luciferase expression. We evaluated the correction of the dystrophin reading frame coupled to luciferase in mice lacking exon 50, a common mutational hotspot, after delivery of CRISPR/Cas9 gene editing machinery with adeno-associated virus. Bioluminescence monitoring revealed efficient and rapid restoration of dystrophin protein expression in affected skeletal muscles and the heart. Our results provide a sensitive non-invasive means of monitoring dystrophin correction in mouse models of DMD and offer a platform for testing different strategies for amelioration of DMD pathogenesis.

## Abstract (web summary)

Dystrophin-deficient mice are used to test corrective strategies for Duchenne muscular dystrophy, but evaluation of dystrophin expression requires collection of tissue samples from specific muscles and time points. Here, the authors generate mice in which dystrophin expression is coupled to luciferase, and show that bioluminescence allows non-invasive monitoring of dystrophin expression following genome editing.

## Introduction

Hundreds of monogenic disorders disrupt the structure and function of striated muscles^1^. Among the most severe muscle diseases is Duchenne muscular dystrophy (DMD), which affects ~1:5000 boys^2^. DMD is typically diagnosed during the first few years of life and inevitably progresses to loss of ambulation by the teenage years and death from respiratory failure and cardiomyopathy in the third decade of life^3^. The disease is caused by mutations in the dystrophin gene, which encodes a large intracellular scaffolding protein that links the cytoskeleton with muscle membranes and is essential for maintenance of muscle integrity^4,5^.

The structures of the muscle dystrophin protein and gene are highly conserved in vertebrate species with 79 exons encoding a protein of 3684 amino acids^2,6^. There are several “hotspot” regions in the gene in which deletions result in splicing of exons that are out of frame, preventing the production of functional dystrophin protein^1,7^. The region that spans exons 45–50 is the most prevalent hotspot region, typically placing exon 51 out of frame with preceding exons and preventing expression of functional dystrophin protein. Therapies that induce “skipping” of exon 51 restore the reading frame, and in principle could benefit approximately 13% of DMD patients^8^.

Numerous therapeutic approaches have been taken to restore muscle function in DMD^9^. To date, the only FDA approved drug for treatment of DMD is eteplirsen, an oligonucleotide that promotes exon 51 skipping^10–14^. Results reported from the clinical trial of 12 patients showed restoration of dystrophin protein expression to less the 1% of normal levels following a year of intravascular infusion of the compound^12,13^. This approach requires life-long drug treatment.

Gene editing with CRISPR/Cas9 to remove or bypass mutations that disrupt the dystrophin open reading frame represents a possible alternative approach to restore dystrophin expression in DMD patients^15–21^. Recently, we demonstrated the efficacy of single-cut genomic editing for restoration of dystrophin expression in mice and human cells harboring a variety of DMD mutations^22^.

A challenge with respect to monitoring possible efficacy of therapies that might restore dystrophin expression in vivo has been the necessity of measuring dystrophin protein in biopsies or sacrificing animals with the disease at specific time points. Thus, it has not been possible to monitor dystrophin expression noninvasively over time in the same animals. In an effort to overcome this challenge, we generated mice in which a luciferase reporter gene was fused in-frame with the endogenous dystrophin gene. In these mice, luciferase bioluminescence serves as a proxy for dystrophin expression. We then introduced an exon 50 deletion into these mice, which extinguished luciferase expression. Using these ΔEx50-Dmd-Luc mice, we were able to optimize the delivery of CRISPR/Cas9 gene editing components using AAV9 for skipping of exon 51 and restoring dystrophin expression. These ΔEx50-Dmd-Luc mice represent a valuable model for optimizing the genetic correction of DMD using CRISPR/Cas9, as well as for studying the impact of other correction strategies and pharmacologic interventions on progression of the disease.

## Results

### Dystrophin–Luciferase reporter mice

**Fig. 1:**
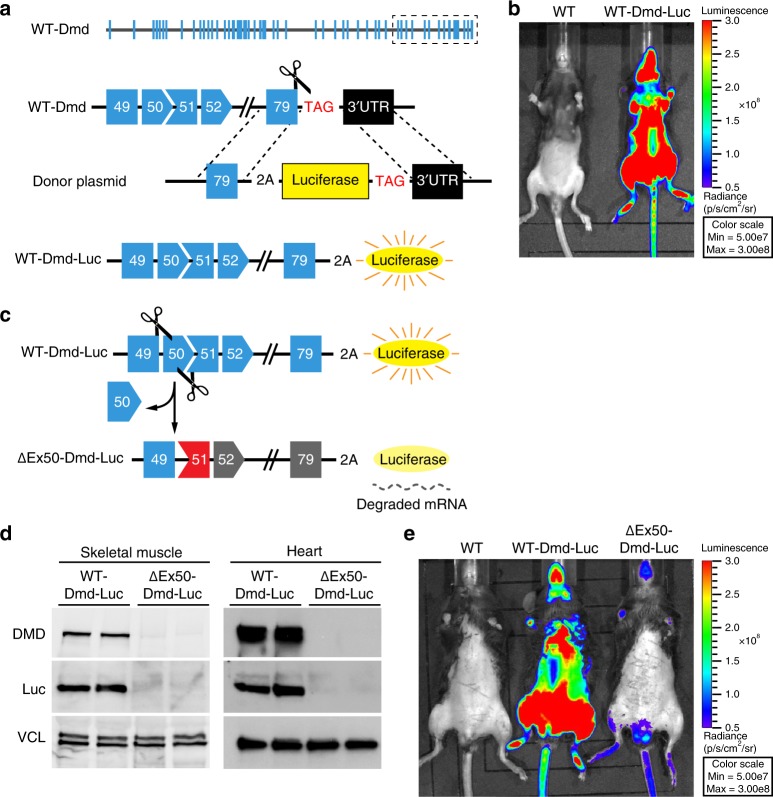
∆Ex50-Dmd-Luc mouse model. **a** Strategy for creation of dystrophin reporter mice. Dystrophin (*Dmd*) gene with exons is indicated in blue. Using CRISPR/Cas9 mutagenesis, we inserted a DNA cassette encoding the luciferase reporter with the protease 2A cleavage site at the 3′ end of the dystrophin coding region. **b** Bioluminescence imaging of wild-type (WT) and *Dmd* knock-in luciferase reporter (referred as WT-Dmd-Luc) mice. **c** Strategy for creation of ΔEx50-Dmd-Luc reporter mice. Dystrophin (*Dmd*) gene with exons is indicated in blue. Using CRISPR/Cas9 mutagenesis, we deleted the exon 50 of *Dmd* gene. **d** Western blot analysis of dystrophin (DMD), luciferase and vinculin (VCL) expression in skeletal muscle and heart tissues. **e** Bioluminescence imaging of wild-type (WT), WT-Dmd-Luc and ΔEx50-Dmd-Luc reporter mice

In an effort to facilitate the analysis of dystrophin correction strategies in vivo in a noninvasive way, reporter mice were generated by insertion of a luciferase expression cassette into the 3′ end of the *Dmd* gene such that luciferase would be translated in-frame with exon 79 of dystrophin (Fig. 1a). To avoid the possibility that luciferase might destabilize the dystrophin protein or perturb its various protein interactions, a protease 2A cleavage site was engineered between the proteins, allowing auto-catalytic cleavage and release from dystrophin after translation (Fig. 1a). The Dmd-luciferase reporter line (WT-Dmd-Luc) was validated by DNA sequencing. Bioluminescence imaging of mice showed high, muscle-specificity of luciferase expression (Fig. 1b).

The most prevalent hot spot region for dystrophin mutations in DMD patients lies between exons 45 and 51 where skipping of exon 51 could potentially correct the largest group of 13–14% of patients^8^. We deleted exon 50 in WT-Dmd-Luc mice using CRISPR/Cas9 with two single-guide RNAs (sgRNAs) to create a reporter line of mice referred to as ∆Ex50*-*Dmd-Luc (Fig. 1c). The deletion of exon 50 was confirmed by DNA sequencing (Supplementary Fig. 1) and placed the dystrophin gene out of frame, preventing dystrophin protein expression in skeletal muscle and heart (Fig. 1d, e and Supplementary Fig. 2). Because expression of luciferase is linked to the translation of dystrophin, the deletion of exon 50 prevents luciferase expression in ∆Ex50-Dmd-Luc mice (Fig. 1d). Residual background bioluminescence detected in these mice is likely attributable to the expression of a smaller isoform of dystrophin (Dp71), which is expressed in tissues other than skeletal muscle^6,23^ and dystrophin isoform Dp116^6^, which is expressed only in Schwann cells. Dp71 and Dp116 isoform expression is initiated from downstream promoters located in intron 62 and intron 55, respectively.

∆Ex50-Dmd-Luc mice showed pronounced dystrophic muscle with necrotic myofibers, fibrosis, and centralized myonuclei, indicative of degeneration and regeneration (Supplementary Fig. 3). Thus, based on the absence of dystrophin protein expression and muscle histology, the ∆Ex50-Dmd-Luc mice represent a faithful model of DMD.

### In vivo noninvasive monitoring of dystrophin correction

To correct the dystrophin reading frame and evaluate the bioluminescence signal in ∆Ex50-Dmd-Luc mice, an sgRNA targeting a region adjacent to the exon 51 splice acceptor site (referred to as sgRNA-51) was used^22^ (Supplementary Fig. 4a). For the in vivo delivery of Cas9 and sgRNA-51 to skeletal muscle and the heart, we used AAV9, which displays preferential tropism for these tissues^24^. Muscle-specific expression of the AAV9-Cas9 vector was further ensured by incorporating the muscle creatine kinase (CK8e) promoter^22,25,26^, which is highly specific for expression in muscle and heart (Supplementary Fig. 4b). Expression of the sgRNA in a separate AAV9 vector was driven by three RNA polymerase III promoters (U6, H1, and 7SK) (Supplementary Fig. 4b)^22^.

**Fig. 2:**
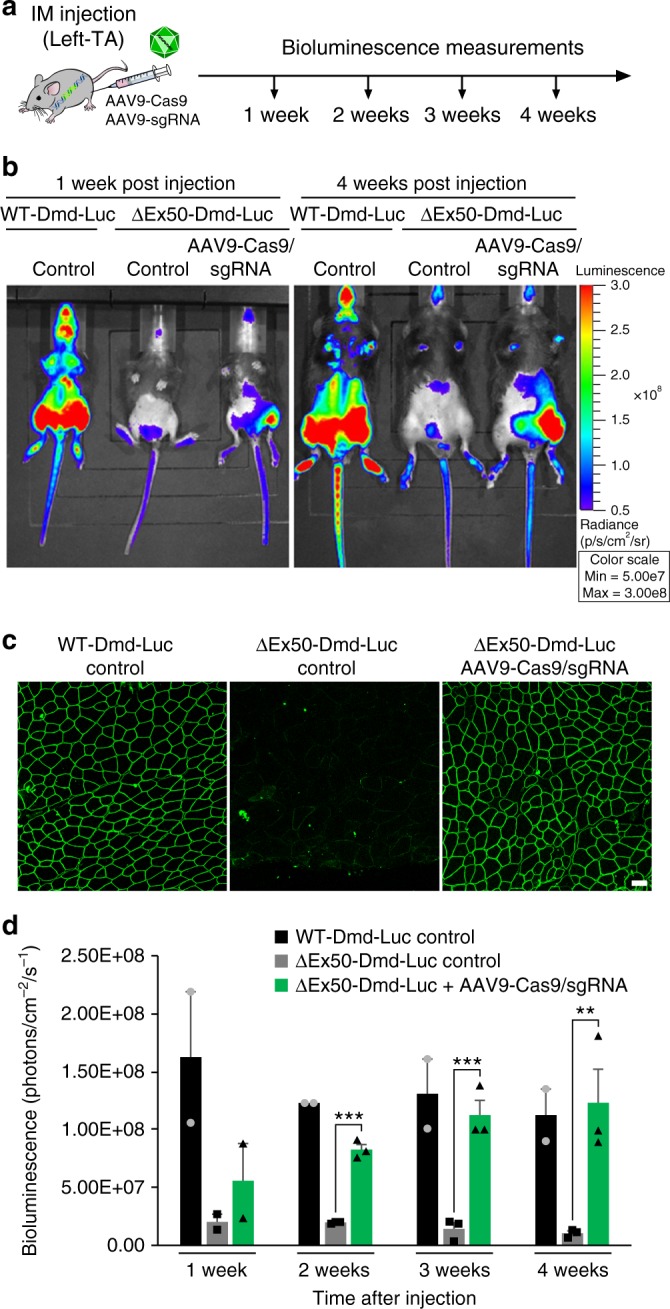
Correction of dystrophin expression by intramuscular injection of AAV9-encoded gene editing components. **a** The left tibialis anterior muscle of ΔEx50-Dmd-Luc mice was injected with AAV9-Cas9 and AAV9-sgRNA. ΔEx50-Dmd-Luc mice were analyzed weekly by bioluminescence. Control mice were injected with saline. **b** Bioluminescence imaging of wild-type (WT), WT-Dmd-Luc and ΔEx50-Dmd-Luc mice injected with AAV9-Cas9 and AAV9-sgRNA 1 week and 4 weeks after injection. **c** Dystrophin immunohistochemistry of tibialis anterior muscle of WT-Dmd-Luc mice, control ΔEx50-Dmd-Luc mice and ΔEx50-Dmd-Luc mice injected with AAV9-Cas9 and AAV9-sgRNA, 4 weeks after injection. **d** Bioluminescent imaging (BLI) measurements of left hindlimb of WT-Dmd-Luc mice, control ΔEx50-Dmd-Luc mice and ΔEx50-Dmd-Luc mice injected with AAV9-Cas9 and AAV9-sgRNA. Data are represented as mean ± SEM. *n* = 3. (^**^P < 0.005, ^***^P < 0.0005). Scale bar: 50 μm

Following intramuscular (IM) injection of the left tibialis anterior (TA) muscle of ∆Ex50-Dmd-Luc mice at postnatal day (P) 12 with 5 × 10^10^ AAV9 viral genomes (vg) of AAV9-Cas9 and 5 × 10^10^ AAV9 viral genomes (vg) of AAV9-sgRNA per TA, muscles were analyzed by dystrophin immunostaining and bioluminescence for 4 weeks (Fig. 2a and Supplementary Fig. 5). Bioluminescence signal was apparent in the injected leg within 1 week after injection and increased in intensity thereafter, ultimately reaching a level comparable to that of WT-Dmd-Luc mice within 4 weeks (Fig. 2b, d and Supplementary Figs. 5–7). Histological analysis of AAV9-injected TA muscle was performed to evaluate the number of fibers that expressed dystrophin and the correlation with the bioluminescence signal. Dystrophin immunohistochemistry of muscle from ∆Ex50-Dmd-Luc mice injected with AAV9-Cas9 and AAV9-sgRNA-51 revealed restoration of dystrophin expression throughout the entire muscle (Fig. 2c, d and Supplementary Fig. 5). In addition, immunohistochemistry and Western blot analysis over time revealed an increase in dystrophin expression at 3 weeks compared to 1 week after injection (Supplementary Figs. 6b, d and 7).

To compare the efficiency of gene editing and dystrophin correction over time, in vivo targeting efficiency was assessed 1 week and 3 weeks after injection within muscle biopsies by tracking indels by decomposition (TIDE) analysis of genomic DNA and reverse transcription polymerase chain reaction (RT-PCR) products with primers for sequences in exons 48 and 53 (Supplementary Fig. 6c). The gene-editing analysis showed a mean of 13% of DNA editing and a mean of 52% of cDNA editing at 1 week after injection and a mean of 14% of DNA editing and a mean of 67% of cDNA editing at 3 weeks after injection. TIDE analysis of genomic DNA and cDNA did not show a significant increase between 1 and 3 weeks, suggesting that gene editing occurs within the first week after injection (Supplementary Fig. 6c). However, due to the large size and long half-life (>100 days)^27^ of the dystrophin protein, it continued to accumulate over time (Supplementary Fig. 6d, e). The luciferase reporter is not fused to the dystrophin protein due to the presence of the 2A self-cleaving peptide. Western blot analysis of luciferase protein expression showed no significant difference between 1 week and 3 weeks, similar to our DNA and cDNA gene-editing results (Supplementary Fig. 6d, f). Therefore, the luciferase reporter gene can serve as a potential indicator of the restoration of the reading frame of the transcript which is achieved within the first week after injection.

**Fig. 3:**
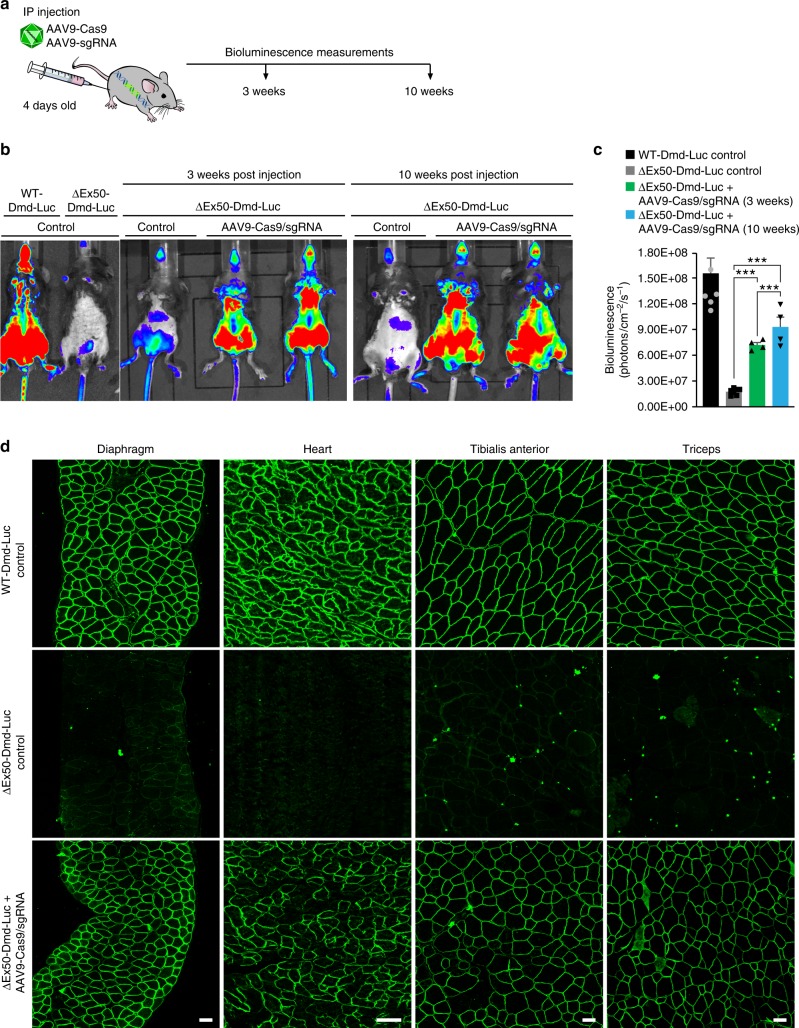
Correction of dystrophin expression by systemic delivery of AAV9-encoded gene editing components. **a** ΔEx50-Dmd-Luc mice were injected intraperitoneally with AAV9-Cas9 and AAV9-sgRNA-51 and analyzed by bioluminescence. Control mice were injected with saline. **b** Bioluminescence imaging of WT-Dmd-Luc mice and ΔEx50-Dmd-Luc mice injected with AAV9-Cas9 and AAV9-sgRNA. **c** Bioluminescent imaging (BLI) measurements of WT-Dmd-Luc mice, control ΔEx50-Dmd-Luc mice and ΔEx50-Dmd-Luc mice injected with AAV9-Cas9 and AAV9-sgRNA. **d** Dystrophin immunohistochemistry of diaphragm, heart, tibialis anterior, and triceps muscles 10 weeks after systemic injection with AAV9-Cas9 and AAV9-sgRNA. Data are represented as mean ± SEM. *n* = 4. (^***^P < 0.0005). Scale bar: 50 μm

**Fig. 4:**
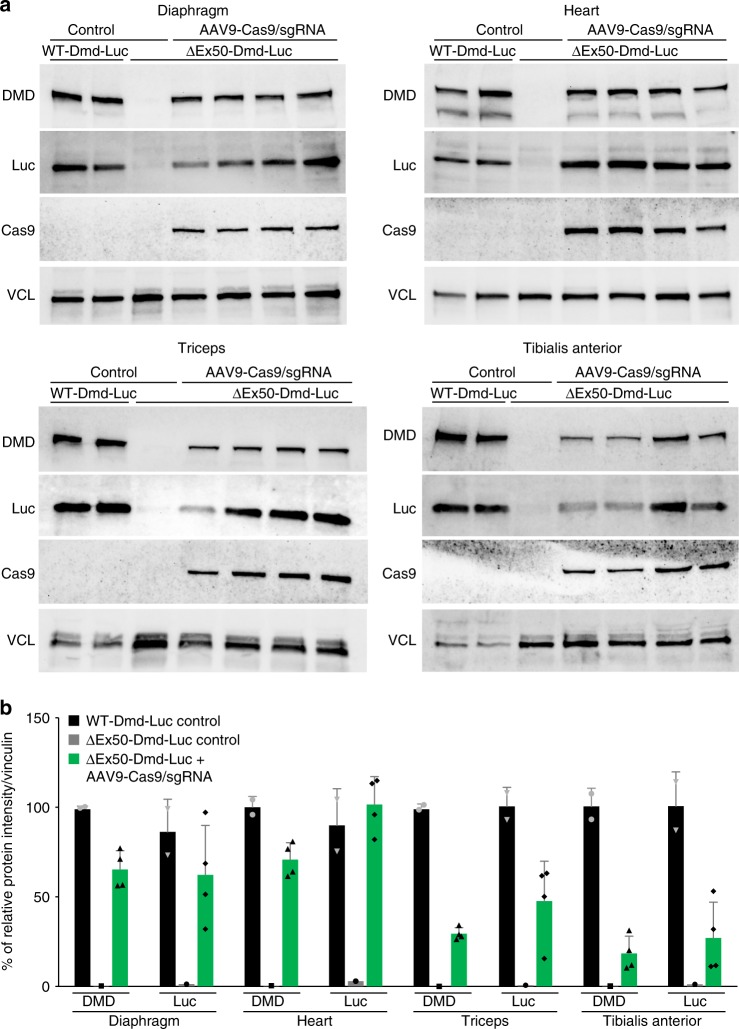
Expression of dystrophin and luciferase following systemic delivery of AAV9-encoded gene editing components. **a** Western blot analysis of dystrophin (DMD), luciferase (Luc), Cas9, and vinculin (VCL) in diaphragm, heart, triceps muscles, and tibialis anterior of WT-Dmd-Luc mice, control ΔEx50-Dmd-Luc mice and ΔEx50-Dmd-Luc mice injected with AAV9-Cas9 and AAV9-sgRNA. **b** Quantification of dystrophin and luciferase expression from blots after normalization to vinculin. Data are represented as mean ± SEM. *n* = 4

To further evaluate the sensitivity of the luciferase reporter in vivo, we administered AAV9-Cas9 and AAV9-sgRNA-51 intraperitoneally to ∆Ex50-Dmd-Luc mice at P4 with 1 × 10^14^ viral genomes/kilogram (vg/kg) of AAV9-Cas9 and 2 × 10^14^ vg/kg of AAV9-sgRNA and luciferase signal was monitored over time (Fig. 3a). Widespread bioluminescence was observed 3 weeks after injection and continued to increase to a level ~70% of WT by 10 weeks. (Fig. 3b, c). Histological analysis and immunohistochemistry revealed widespread dystrophin expression in the diaphragm, heart, TA, and triceps muscles of gene-edited ΔEx50-Dmd-Luc mice at 10 weeks post injection (Fig. 3d and Supplementary Fig. 8). H&E staining of multiple skeletal muscles showed that histopathologic hallmarks of muscular dystrophy, such as necrotic myofibers, were also largely corrected 10 weeks after delivery of AAV9-Cas9 and AAV9-sgRNA-51 (Supplementary Fig. 9). Western blot analysis revealed a correlation between expression of Cas9, dystrophin, and luciferase in skeletal muscle and heart following systemic IP delivery of AAV9-encoded gene-editing components to ΔEx50-Dmd-Luc mice (Fig. 4). To confirm the specificity of the CK8e muscle promoter used for AAV9 delivery of Cas9, we analyzed Cas9 protein expression in skeletal muscles, heart and liver tissues 10 weeks after systemic injection. Western blot analysis showed expression of Cas9 only in muscle and heart tissue and no expression in liver 10 weeks after injection (Supplementary Fig. 10).

In vivo targeting efficiency was assessed within muscle biopsies by TIDE analysis of RT-PCR products with primers for sequences in exons 48 and 53 (Supplementary Fig. 11). TIDE analysis showed 68.6%, 87.08%, 29.6% and 66.5% of indels for diaphragm, heart, TA, and triceps muscle, respectively (Supplementary Fig. 11). To investigate the proportions of various indels generated by systemic delivery of AAV9-Cas9 and AAV9-sgRNA-51, we performed amplicon deep-sequencing analysis of the genomic DNA and cDNA from diaphragm muscle 10 weeks after injection. Genomic deep-sequencing analysis revealed a consistent predominance of a single nucleotide insertion 3′ to the PAM sequence that reframes the *Dmd* transcript in all treated mice, representing 9–10% of indels (Supplementary Fig. 12). Similarly, deep sequencing of cDNA products revealed that 50–63% of total reads contained predominant reframed cDNA products with a single-nucleotide insertion, while 26–39% contained nonedited cDNA product (Supplementary Fig. 12).

## Discussion

The ∆Ex50-*Dmd*-Luc reporter mice described in this study resolve a major challenge associated with the analysis of DMD therapies in mice^28,29^. Because documentation of dystrophin production in vivo necessitated sacrificing animals at different time points, it has not been possible to monitor the impact of therapies over time in the same animal. The WT-Dmd-Luc mice allow the detection of dystrophin expression with high sensitivity and specificity. While we have focused here on the correction of an exon 50 deletion on the restoration of dystrophin expression by AAV9-mediated delivery of gene-editing components in vivo, the same approach can be taken for monitoring any other DMD mutations. The WT-Dmd-Luc mice will also be useful for investigating the impact of other DMD therapies either individually or in combination with CRISPR/Cas9-mediated editing.

At 10 weeks post injection, comparison of dystrophin protein production by Western blot analysis or immunohistochemistry showed a correlation with luciferase expression from the reporter gene, validating the luciferase reporter as an indicator for restoration of the reading frame of the endogenous dystrophin gene. At early time points such as 1 week and 3 weeks after expression, the correlation of luciferase and dystrophin protein expression is challenging and not linear due to differences in protein size and half-lives of dystrophin (427 kDa) and luciferase (24 kDa). Since the luciferase protein is not fused to the dystrophin protein due to the presence of the 2A self-cleaving peptide, luciferase expression serves as an indicator of the restoration of the reading frame of the transcript which is achieved within the first week after injection. Interestingly, DNA and cDNA editing analysis suggested that CRISPR/Cas9-mediated editing occurs within the first week in vivo, whereas the dystrophin protein continues to be expressed and increases over time. Although this study does not demonstrate a direct linear relationship between DMD expression and luciferase expression, it provides a sensitive assay for noninvasive detection of dystrophin gene editing. Future experiments will be needed to determine if and to which extent this model can be used to optimize therapies.

Given the many variables involved in achieving optimal restoration of dystrophin expression in vivo through AAV9-mediated delivery of gene editing components, including viral titer, vector design, time course of AAV delivery, influence of combination therapies and differing responses of cardiac and skeletal muscles, these luciferase reporter mice should accelerate the preclinical development of CRISPR/Cas9 editing for correction of DMD mutations.

## Materials and methods

### Study approval

All experimental procedures involving animals in this study were reviewed and approved by the University of Texas Southwestern Medical Center’s Institutional Animal Care and Use Committee.

### Mice

Mice were housed in a barrier facility with a 12-h light/dark cycle and maintained on standard chow (2916 Teklad Global). To generate WT-Dmd-luciferase mice and introduce the luciferase gene into the Dmd locus, a sgRNA specific to the exon 79 sequence of the mouse *Dmd* locus was cloned into vector px330 using the primers from Supplementary Table 1. A donor vector containing the protease 2A and luciferase reporter sequence was constructed by incorporating short 5′ and 3′ homology arms specific to the *Dmd* gene locus and used as a template for CRISPR/Cas9-mediated homologous recombination.

To generate ∆Ex50-Dmd-Luc mice 2 sgRNA specific intronic regions surrounding exon 50 sequence of the mouse *Dmd* locus were cloned into vector px330 using the primers from Supplementary Table 1. For the in vitro transcription of sgRNA, T7 promoter sequence was added to the sgRNA template by PCR using the primers from Supplementary Table 1. The gel purified PCR products were used as template for in vitro transcription using the MEGAshortscript T7 Kit (Life Technologies). sgRNA was purified by MEGAclear kit (Life Technologies) and eluted with nuclease-free water (Ambion). The concentration of sgRNA was measured by a NanoDrop instrument (Thermo Scientific).

### Genotyping of Dmd-Luc and ∆Ex50-Dmd*-*Luc mice

WT-Dmd-Luc and ∆Ex50-Dmd-Luc mice were genotyped using primers encompassing the targeted region shown in Supplementary Table 1. Tail biopsies were digested in 100 μL of 25-mM NaOH, 0.2-mM EDTA (pH 12) for 20 min at 95 °C. Tails were briefly centrifuged followed by addition of 100 μL of 40-mM Tris⋅HCl (pH 5) and mixed to homogenize. Two microliters of this reaction were used for subsequent PCR reactions with the primers below, followed by gel electrophoresis.

### Plasmids

The pSpCas9(BB)-2A-GFP (PX458) plasmid containing the human codon optimized SpCas9 gene with 2A-EGFP and the backbone of sgRNA was purchased from Addgene (Plasmid #48138). Cloning of sgRNA was performed using Bbs I site.

### Cas9 plasmid and sgRNA assembly in the AAV9 backbone

The AAV9-CK8-Cas9 vector has been previously described^22^. Cloning of sgRNA in three copies under transcriptional control of three different promoters U6, H1, or 7SK has also been previously described^22^. To test for correct assembly, the plasmid was sequenced using the primer Dono-R-5′-GTATGTTGTGTGGAATTGTGAG-3′.

### AAV9 strategy and delivery to ΔEx50-Dmd-Luc mice

*Dmd* exon 51 sgRNAs sequences were cloned using primers in Supplementary Table S1. Cloning of sgRNA was done using a Bbs I site. Assembly of the AAV9 backbone cloning system relies on two consecutive steps of the Golden Gate Assembly (New England Biolabs). In the first step of assembling the sgRNA into the donor plasmid, annealing of oligonucleotides was performed by heating a reaction containing 2.5 μl of each oligo (0.5 μM), 5 μl of NEBuffer 2 (NEB), and 40 μl of ddH_2_O to 95 °C for 5 min using a heating block. For the assembly reaction into the donor plasmid, 40 fmol (~100 ng) of destination backbone was mixed with 1 μl of annealed, diluted oligos, 0.75 μl of Esp3I (Thermo Scientific), 1 μl of buffer tango (Thermo Scientific), 1 μl of T4 DNA ligase (400 U/μl) (NEB), and ATP (adenosine 5′-triphosphate), and DTT (dithiothreitol) at a final concentration of 1 μM in 10-μl total volume. Using a thermo- cycler, PCR was performed for 25–50 cycles at 37 °C for 3 min followed by 20 °C for 5 min. Restriction enzyme and ligase were then denatured by heating to 80 °C for 20 min. Three microliters of this reaction was used for transformation of chemocompetent bacteria, which were recovered in super optimal broth with catabolite repression (SOC) (37 °C, 800 g, 40 min) and spread on LB agar plates containing chloramphenicol (25 mg/ml). Annealed oligonucleotides encoding for the sgRNA were cloned into donor plasmids that carry the negative selection marker ccdB (to reduce background during cloning) and the chloramphenicol resistance gene.

To test for correct assembly, the plasmid was sequenced using the primer Dono-R-5′-GTATGTTGTGTGGAATTGTGAG-3′. In the second step, three of these donor plasmids driving the expression of one sgRNA under transcriptional control of the U6, H1, or 7SK promoter were pooled in a second Golden Gate Assembly along with a recipient plasmid that carries AAV9 inverted terminal repeats (ITRs). The assembly reaction contained all four plasmids: donor plasmid-#1-U6-sgRNA, donor plasmid-#2-H1-sgRNA, donor plasmid-#3–7SK-sgRNA, and recipient plasmid containing the ITR. Digestion with Bbs I generated unique overhangs for each fragment (U6, H1, 7SK, and recipient backbone). During the ligation procedure, these overhangs annealed; a circularized plasmid was only obtained when the three cassettes matched each other.

Prior to AAV9 injections, ΔEx50-Dmd-Luc mice were anesthetized by intraperitoneal (IP) injection of ketamine and xylazine anesthetic cocktail. For IM injection, TA muscle of P12 male ∆Ex50-Dmd-Luc mice was injected with 50 µl of AAV9 (10^12^ vg/ml) preparations. For IP injection, P4 ∆Ex50-Dmd-Luc mice were injected using an ultrafine needle (31 G) with 80 µl of 1 × 10^14^ vg/kg for AAV9-Cas9 and 2 × 10^14^ vg/kg AAV9-sgRNA.

### Bioluminescence imaging

Bioluminescence imaging was performed using the IVIS Spectrum Imaging System from Xenogen (Caliper Life Sciences). The hair was removed using Nair hair removal lotion prior to imaging. The mice were anesthetized using 2% isoflurane and 100% Oxygen with a flow rate of 2.5 L/min. Sterile d-luciferin at a concentration of 40 mg/mL was administered by IP injection at 100 μL per mouse. Up to 12 images were collected for 30 s at the maximum light collection. The images were saved for analysis. The image analysis was performed using Living Image 4.5.2 (Caliper Life Sciences). A manually generated circle (ROI function) was placed upon the region of interest of the mouse. Bioluminescence values are indicated as radiance (photons/cm^−2^/s^−1^).

### Histological analysis of muscles

Histological analysis of muscles was performed as described previously^22^. In brief, muscle cryoembedded in a 1:2 volume mixture of Gum Tragacanth powder (G1128, Sigma-Aldrich) were snap frozen in isopentane supercooled to −155 °C. Resulting blocks were stored at −80 °C prior to sectioning. Eight-micrometer transverse sections of the skeletal muscle were prepared on a Leica CM3050 cryostat and air dried prior to staining on the same day. H&E staining was performed according to established staining protocols^14^, and dystrophin immunohistochemistry was performed using MANDYS8 monoclonal antibody (D8168, Sigma-Aldrich). Cryostat sections were thawed and rehydrated/delipidated in 1% Triton/phosphate-buffered saline, pH 7.4 (PBS). Following delipidation, sections were washed free of Triton, incubated with mouse immunoglobulin G (IgG) blocking reagent (M.O.M. Kit, Vector Laboratories), washed, and sequentially equilibrated with M.O.M. protein concentrate/PBS, and MANDYS8 diluted 1:1000 in M.O.M. protein concentrate/PBS. Following overnight primary antibody incubation at 4 C, sections were washed, incubated with M.O.M. biotinylated anti-mouse IgG (diluted 1:250), washed, and detection completed with incubation of Vector fluorescein-avidin DCS (diluted 1:250). Nuclei were counterstained with propidium iodide (Molecular Probes) prior to cover slipping with Vectashield.

### Western blot analysis

For Western blot of skeletal or heart muscles, tissues were crushed into fine powder using a liquid nitrogen-frozen crushing apparatus. Tissue lysates were passed (10 times) through a 25-gauge syringe and then (10 times) through a 27-gauge syringe. Protein concentration was determined by BCA assay, and 50 μg of total protein was loaded onto a 4–20% acrylamide gel. Gels were run at 100 V for 15 min and switched to 120 V for 3 h, followed by a 1-h 20-min transfer to a polyvinylidene difluoride membrane at 100 V at 4 °C. The blot was incubated with mouse antidystrophin antibody (1:1000, D8168, Sigma-Aldrich), mouse anti-Cas9 antibody (1:1000, Clone 7A9, Millipore, MAC133), vinculin (1:1000, V9131, Sigma-Aldrich), luciferase (1:1000, Ab21176, Abcam) at 4 °C overnight, and then with goat anti-mouse horseradish peroxidase (HRP) antibody or goat anti-rabbit HRP antibody (1:8000, Bio-Rad Laboratories) at room temperature for 1 h. The blot was developed using Western Blotting Luminol Reagent (Santa Cruz, sc-2048). Full uncropped and unprocessed scans are illustrated in Supplementary Figs. 13–21.

### TIDE analysis

In the first step of TIDE, RT-PCR products around the editing site from muscles were generated using primers designed against the respective target region (Supplementary Table 1). The PCR products were then directly subjected to sequencing. The sequencing results were analyzed using TIDE software package (http://tide.nki.nl). TIDE first aligns the sgRNA sequence to the control sequence to determine the position of the expected Cas9 break site. Then, the control sequence region upstream of the break site is aligned to the experimental sample sequence in order to determine any offset between the two sequence reads. Alignments were done using standard Smith–Waterman local alignment implemented in the *BioStrings* package in Bioconductor. The software uses the peak heights for each base, as determined by the sequence analysis software using 3730 Series Data Collection Software V4 and Sequencing Analysis Software V6. TIDE uses these peak heights to determine the relative abundance of aberrant nucleotides over the length of the whole-sequence trace. An overview of TIDE algorithm and output has been previously described^30^.

### Targeted DNA deep sequencing

PCR of genomic DNA and cDNA from muscles was performed using primers (listed in Supplementary Table S1) designed against the respective target region. A second round of PCR was used to add Illumina flowcell binding sequences and experiment-specific barcodes on the 5′ end of the primer sequence (Supplementary Table S1). Before sequencing, DNA libraries were analyzed using a Bioanalyzer High Sensitivity DNA Analysis Kit (Agilent). Library concentration was then determined by qPCR using a KAPA Library Quantification Kit for Illumina platforms. Library concentration was then determined by qPCR using a KAPA Library Quantification Kit for Illumina platforms. The resulting PCR products were pooled and sequenced with 300 bp paired-end reads on an Illumina MiSeq instrument. Samples were demultiplexed according to assigned barcode sequences. FASTQ format data was analyzed using the CRISPResso software. The alignment of reads at the cleavage site was further analyzed and regrouped in the nonedited, single-nucleotide insertion (+1N), in frame insertion, out of frame insertion, and deletion groups.

### Statistics

Values are presented as mean ± S.E.M. Differences between respective groups were assessed using unpaired two-tailed Student’s *t* tests. *P* < 0.05 was regarded as significant. Statistical analysis was performed in Excel (Microsoft).

## Supplementary information

Supplementary Information

## Data Availability

The data sets generated and/or analyzed in the current study are available from corresponding author on request.
