# Supplementary material for: In vivo non-invasive monitoring of dystrophin correction in a new Duchenne muscular dystrophy reporter mouse
Source: Nat Commun. 2019 Oct 4;10:4537. doi: 10.1038/s41467-019-12335-x (PMC6778191; doi:10.1038/s41467-019-12335-x)
Supplement: Supplementary file 1 — Supplementary Information [file 41467_2019_12335_MOESM1_ESM.pdf]

Supplementary Information

In vivo non-invasive monitoring of dystrophin correction in a new Duchenne Muscular Dystrophy reporter mouse

Leonela Amoasii[1,2], Hui Li[1], Yu Zhang[1], Yi-Li Min[1,2], Efrain Sanchez-Ortiz[1], John M. Shelton[3], Chengzu Long[1],[4], Alex A. Mireault[1], Samadrita Bhattacharyya[1], John R. McAnally[1], Rhonda Bassel-Duby[1], Eric N. Olson[1**]

Department of [1]Molecular Biology, [1]Hamon Center for Regenerative Science and Medicine, [1]Sen. Paul D. Wellstone Muscular Dystrophy Cooperative Research Center, and [2]Exonics Therapeutics, 490 Arsenal Way, Watertown, MA 02472, [3]Department of Internal Medicine, University of Texas Southwestern Medical Center, 5323 Harry Hines Boulevard, Dallas, TX 75390 USA.

[4]Current address: Leon H. Charney Division of Cardiology, New York University School of Medicine, New York, NY 10016, USA

***Correspondence to:** E-mail: Eric.Olson@utsouthwestern.edu

Supplementary Figures: 21

Supplementary Tables: 1

Supplementary Figures

```
WT-Dmd-Luc        GAAATCACTTTTTACCTGTAATTATTCATGTTTCTCATAAAGAATATACTCAGAAAATGA
ΔEx50-Dmd-Luc     GAAATCACTTTTTACCTGTAATTATTCATGTTTCTCATAAAGAATATACTCAGAAAATGA
                  ************************************************************

                        sgRNA-#1-target sequence  PAM
WT-Dmd-Luc        TATTAAAATGATGAGTGAAGTTATATTGGCAATGTTTGTAAAATCAGTAACACGTATGCT
ΔEx50-Dmd-Luc     TATTAAAATGATGAGTGAAGTTA-------------------------------------
                     ***********************

WT-Dmd-Luc        TTCCTATTAAAGAGGAAGTTAGAAGATCTGAGGTCTGAGTGGGAGGCTGTAAACCATTTA
ΔEx50-Dmd-Luc     ------------------------------------------------------------

WT-Dmd-Luc        CTTCGGGAGCTGAGGACAAAGCAGCCTGACCGTGCCCCTGGACTGAGCACTACTGGAGCC
ΔEx50-Dmd-Luc     ------------------------------------------------------------

                                                            sgRNA-#2-target sequence
WT-Dmd-Luc        TGTAAGTACATTTTGTCCCAGGTGTTCTCTTTTGCTTTAGCTGTTTGTTCAAAAGCGTGG
ΔEx50-Dmd-Luc     ----------------------------------------------------------GG
                                                                            **

                    PAM
WT-Dmd-Luc        CTGGGAGGTTATCTCTGAGTGAAAGGAAAATTGGTTTCTGATTCTGCATAAAGGAGTAAA
ΔEx50-Dmd-Luc     CTGGGAGGTTATCTCTGAGTGAAAGGAAAATTGGTTTCTGATTCTGCATAAAGGAGTAAA
                  ************************************************************
```

Supplementary Figure 1 ΔEx50-Dmd-Luc mouse model analysis. Genomic sequence of targeted locus of WT-Dmd-Luc (top line) and ΔEx50-Dmd-Luc founder (bottom line) with a 215 base pair deletion that eliminated exon 50 (indicated in color green). sgRNA-#1 and #2 are indicated in blue.

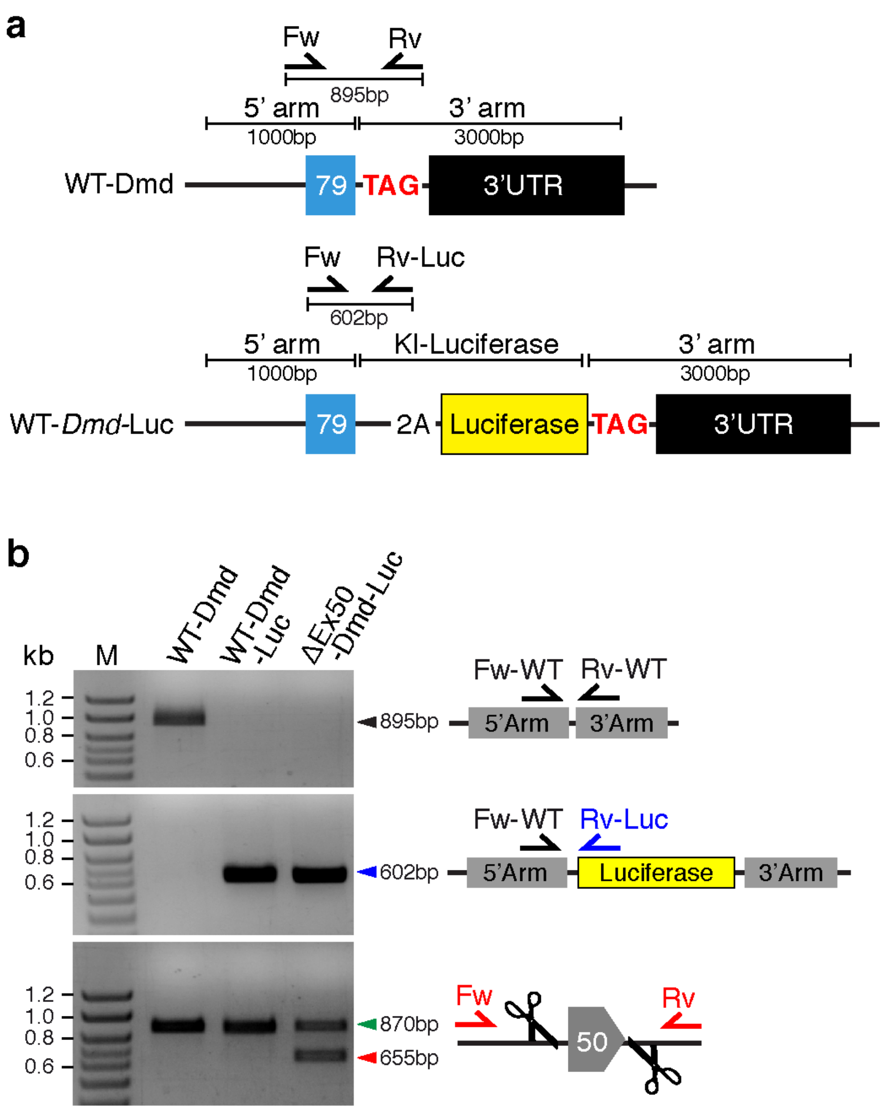

Supplementary Figure 2 ΔEx50-Dmd-Luc reporter mouse genotyping strategy. (**a**) Illustration of the WT-Dmd-Luc and WT-Dmd-Luc genes. (**b**) Genotyping results of ΔEx50-Dmd-Luc reporter mice. Schematic of genotyping strategy and forward (Fw) and reverse (Rv) primers. The ΔEx50-Dmd-Luc mouse is the female founder of the colony.

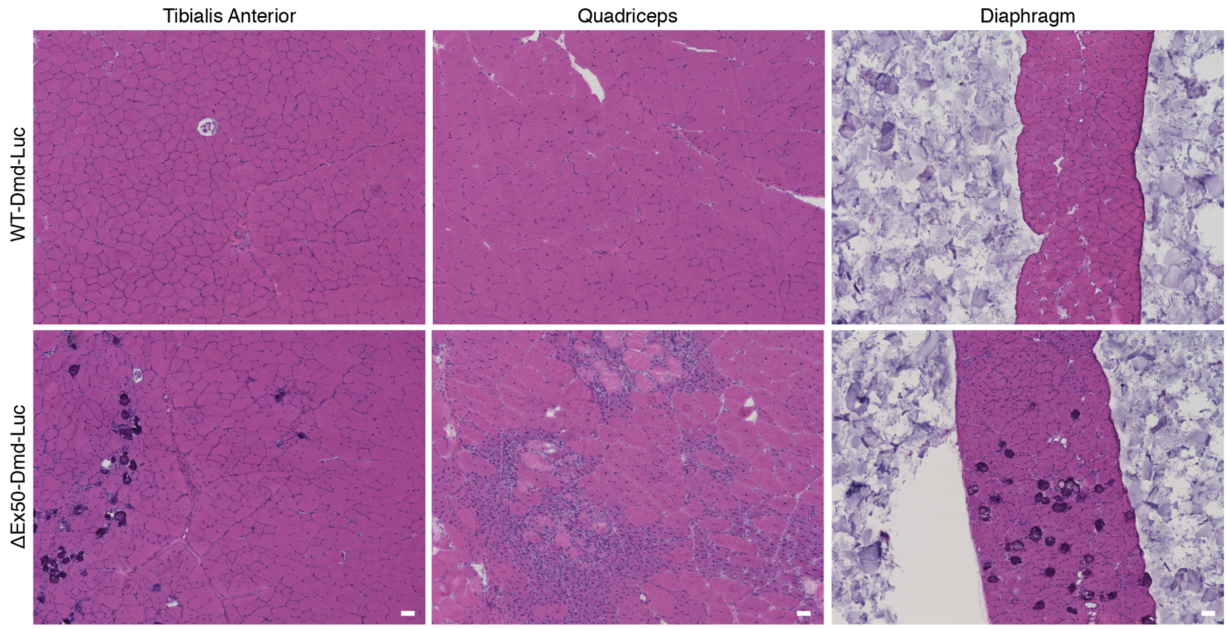

Supplementary Figure 3 Muscle histological analysis of the ΔEx50-Dmd-Luc mouse model. Hematoxylin and eosin (H&E) staining of tibialis anterior, quadriceps and diaphragm muscles of 6 week old WT-Dmd-Luc and ΔEx50-Dmd-Luc mice. n=5. Scale bar: 50μm.

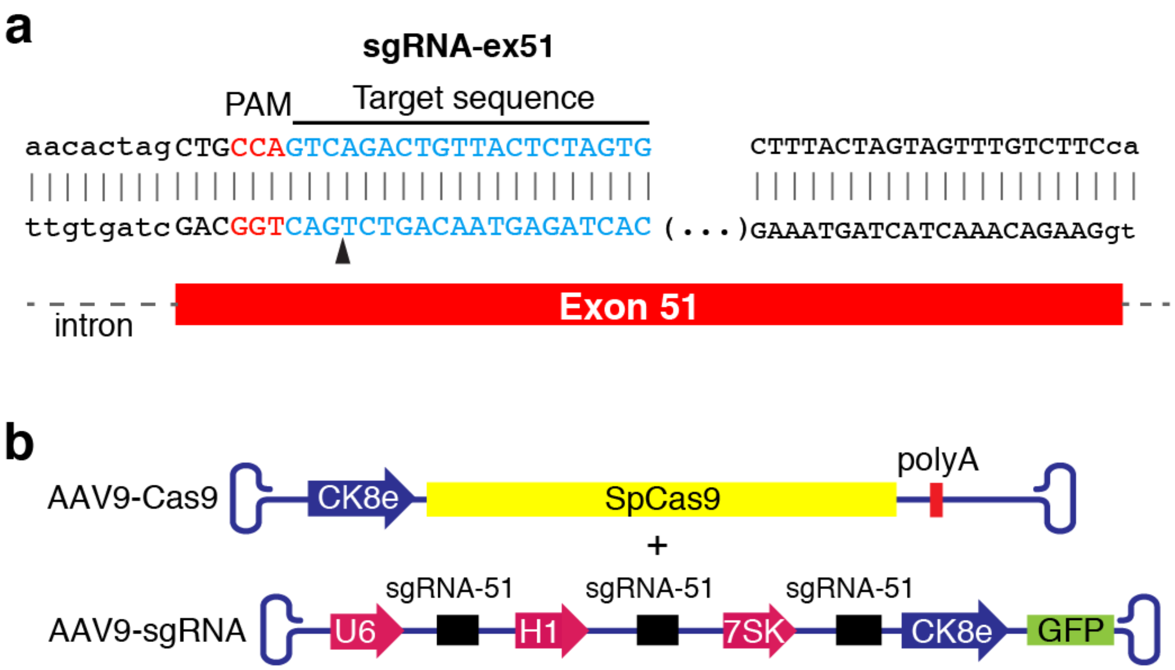

Supplementary Figure 4 Strategy for CRISPR/Cas9-mediated genome editing in ΔEx50 mice. (**a**) Illustration of sgRNA binding position and sequence for sgRNA-ex51. PAM sequence for sgRNA is indicated in red. Black arrow indicates the cleavage site. (**b**) The muscle creatine kinase 8 (CK8e) promoter was used to express SpCas9. The U6, H1 and 7SK promoters for RNA polymerase III were used to express sgRNAs.

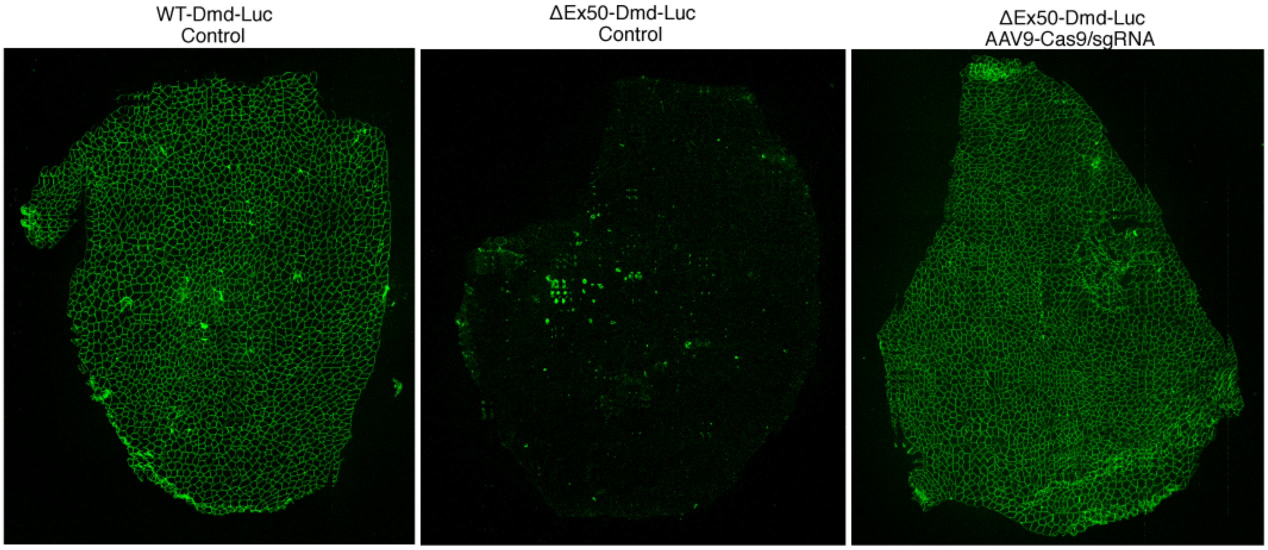

Supplementary Figure 5 Dystrophin immunohistochemistry of entire tibialis anterior muscle of WT-Dmd-Luc mice, control ΔEx50-Dmd-Luc mice and ΔEx50-Dmd-Luc mice injected with AAV9-Cas9 and AAV9-sgRNA, 4 weeks after injection.

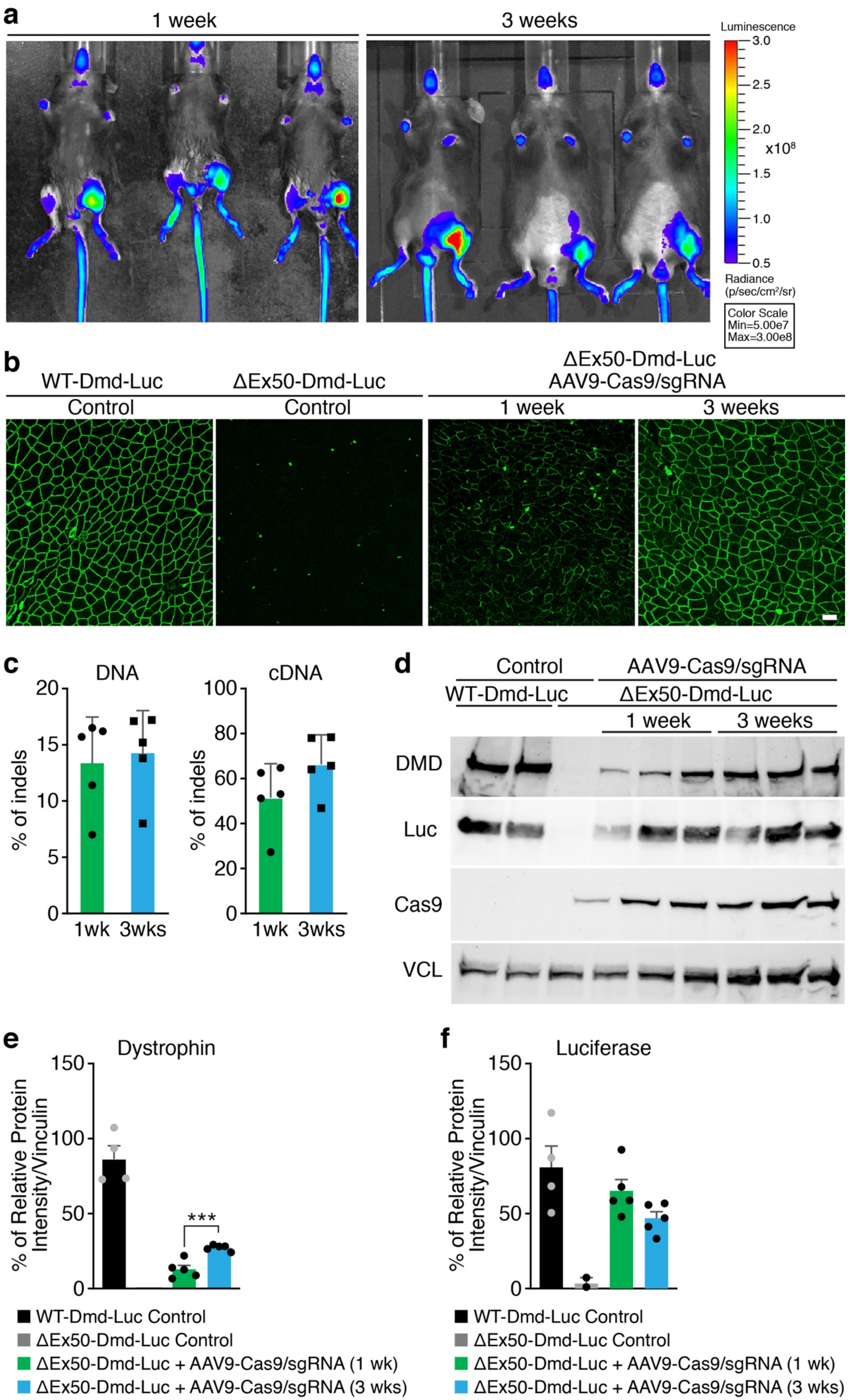

a
1 week
3 weeks
Luminescence
3.0
2.5
2.0
x10^8
1.5
1.0
0.5
Radiance (p/sec/cm²/sr)
Color Scale Min=5.00e7 Max=3.00e8
b
WT-Dmd-Luc
Control
ΔEx50-Dmd-Luc
Control
ΔEx50-Dmd-Luc AAV9-Cas9/sgRNA
1 week
3 weeks
c
DNA
% of indels
1wk 3wks
cDNA
% of indels
1wk 3wks
d
Control
AAV9-Cas9/sgRNA
WT-Dmd-Luc
ΔEx50-Dmd-Luc
1 week
3 weeks
DMD
Luc
Cas9
VCL
e
Dystrophin
% of Relative Protein Intensity/Vinculin
***
WT-Dmd-Luc Control
ΔEx50-Dmd-Luc Control
ΔEx50-Dmd-Luc + AAV9-Cas9/sgRNA (1 wk)
ΔEx50-Dmd-Luc + AAV9-Cas9/sgRNA (3 wks)
f
Luciferase
% of Relative Protein Intensity/Vinculin
WT-Dmd-Luc Control
ΔEx50-Dmd-Luc Control
ΔEx50-Dmd-Luc + AAV9-Cas9/sgRNA (1 wk)
ΔEx50-Dmd-Luc + AAV9-Cas9/sgRNA (3 wks)

Supplementary Figure 6 Correction of dystrophin expression by intra-muscular injection of AAV9-encoded gene editing components. (**a**) Bioluminescence imaging of ΔEx50-Dmd-Luc mice injected with AAV9-Cas9 and AAV9-sgRNA 1 week and 3 weeks after injection. (**b**) Dystrophin immunohistochemistry of tibialis anterior muscle of WT-Dmd-Luc mice, control ΔEx50-Dmd-Luc mice and ΔEx50-Dmd-Luc mice injected with AAV9-Cas9 and AAV9-sgRNA, 1 week and 3 weeks after injection. (**c**) Percentage of indels detected at exon 51 after AAV9-Cas9 and AAV9-sgRNA-51 treatment using tracking indels by decomposition (TIDE) analysis of genomic DNA and RT-PCR (cDNA) products from tibialis anterior ΔEx50-Dmd-Luc mice injected with AAV9-Cas9 and AAV9-sgRNA-51 1 week and 3 weeks after injection. (**d**) Western blot analysis of dystrophin (DMD), luciferase (Luc), Cas9 and vinculin (VCL) in tibialis anterior of WT-Dmd-Luc mice, control ΔEx50-Dmd-Luc mice and ΔEx50-Dmd-Luc mice injected with AAV9-Cas9 and AAV9-sgRNA. (**e**) Quantification of dystrophin expression from blots after normalization to vinculin. (**f**) Quantification of luciferase expression from blots after normalization to vinculin. Data are represented as mean ± SEM. n=5. (***$P<0.0005$). Scale bar: 50μm.

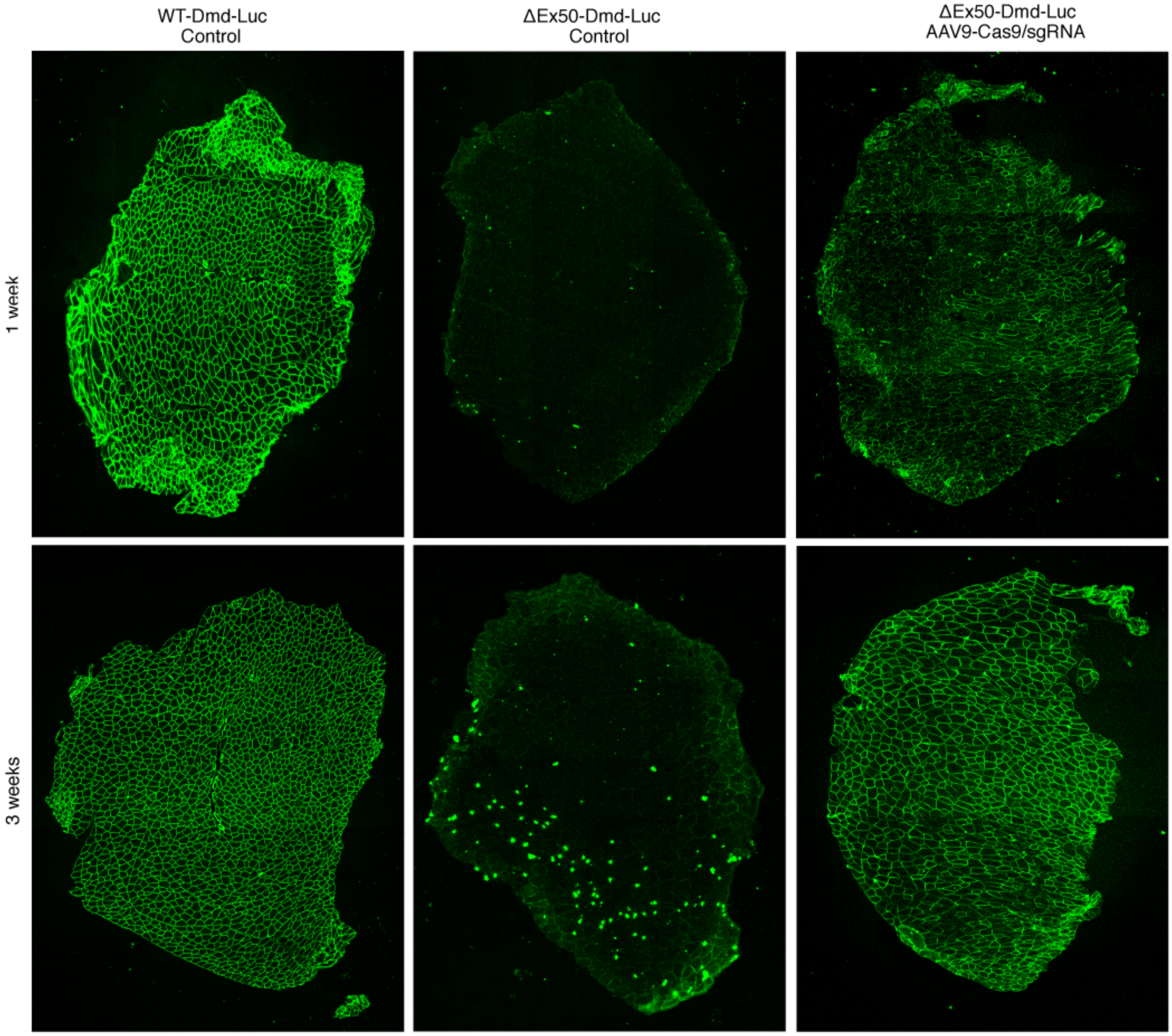

Supplementary Figure 7 Dystrophin immunohistochemistry of entire tibialis anterior muscle of WT-Dmd-Luc mice, control ΔEx50-Dmd-Luc mice and ΔEx50-Dmd-Luc mice injected with AAV9-Cas9 and AAV9-sgRNA, 1 week and 3 weeks after injection.

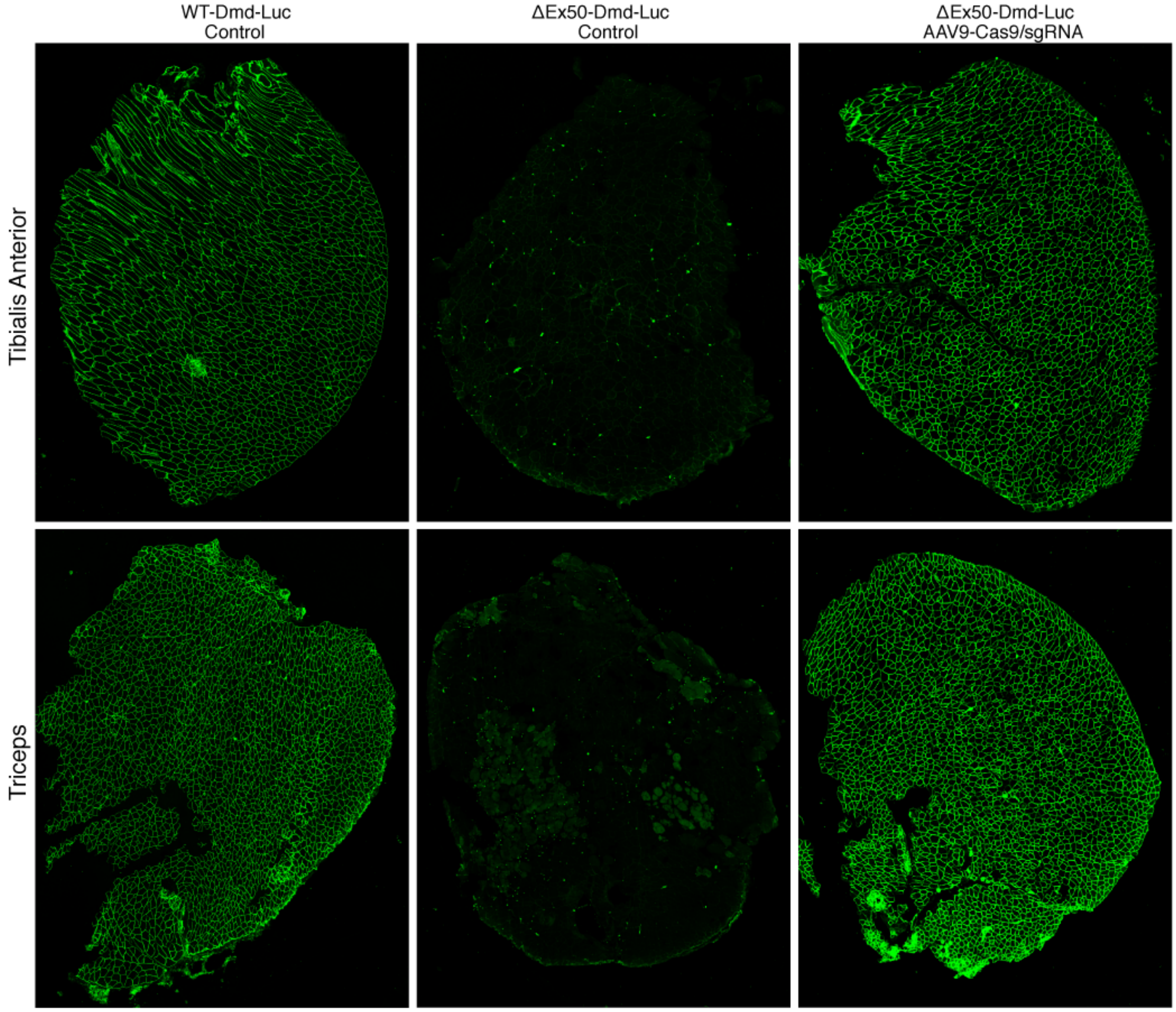

Supplementary Figure 8 Dystrophin immunohistochemistry of entire tibialis anterior and triceps muscles of WT-Dmd-Luc mice, control ΔEx50-Dmd-Luc mice and ΔEx50-Dmd-Luc mice injected with AAV9-Cas9 and AAV9-sgRNA, 10 weeks after injection.

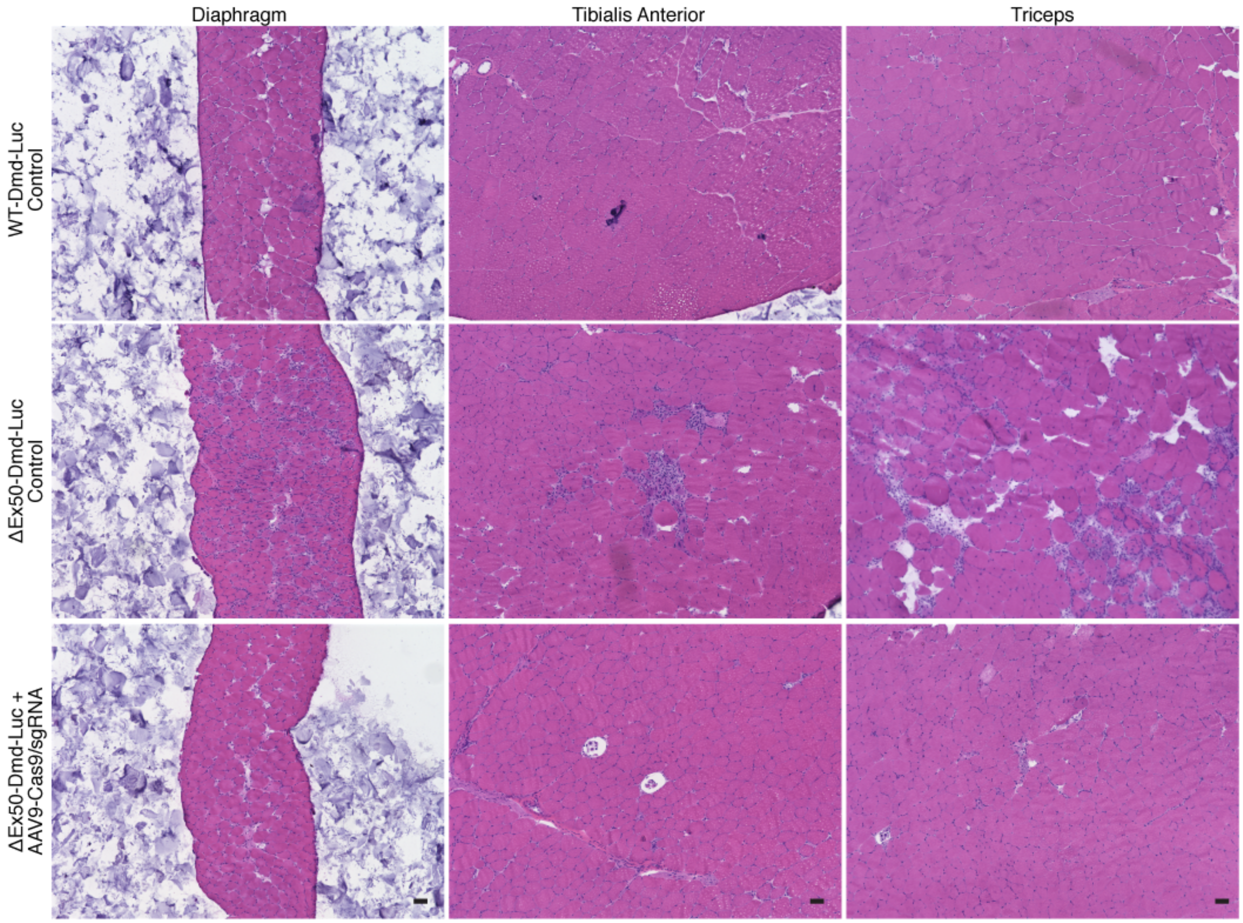

Supplementary Figure 9 Muscle histological analysis of the ΔEx50-Dmd-Luc mice 10 weeks after systemic injection. Hematoxylin and eosin (H&E) staining of diaphragm, tibialis anterior and triceps muscles. n=4. Scale bar: 50μm.

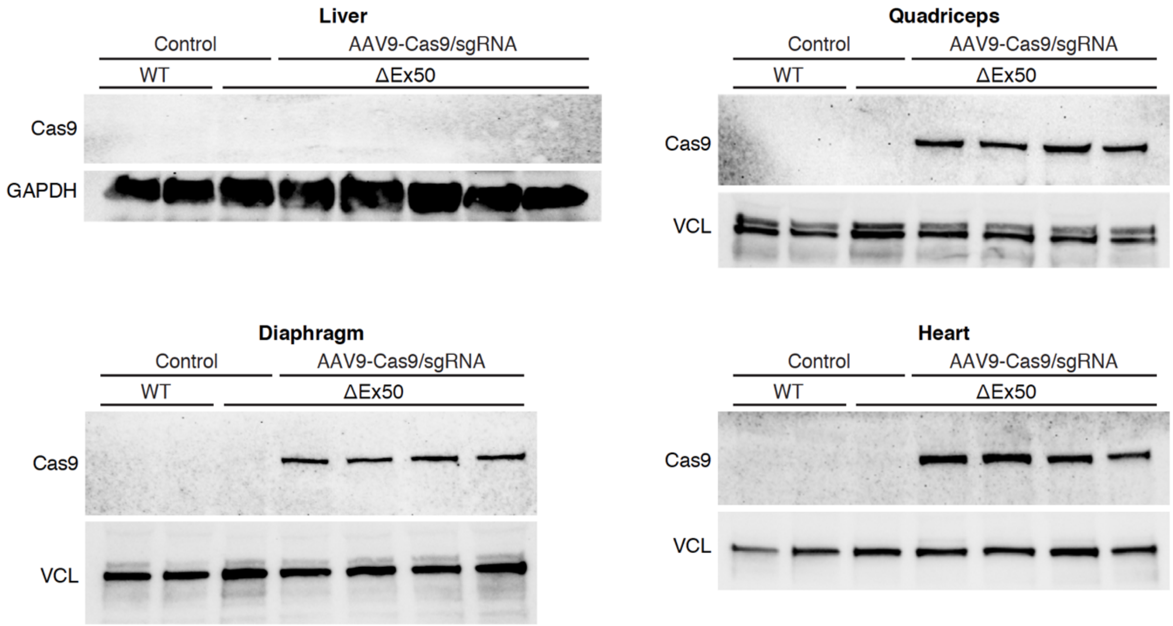

Supplementary Figure 10 Cas9 protein expression 10 weeks following systemic delivery of AAV9-encoded gene editing components. Western blot analysis of Cas9 and vinculin (VCL) in liver, quadriceps, diaphragm and heart of ΔEx50-Dmd-Luc mice 10 weeks after systemic injection with AAV9-Cas9 and AAV9-sgRNA. n=4.

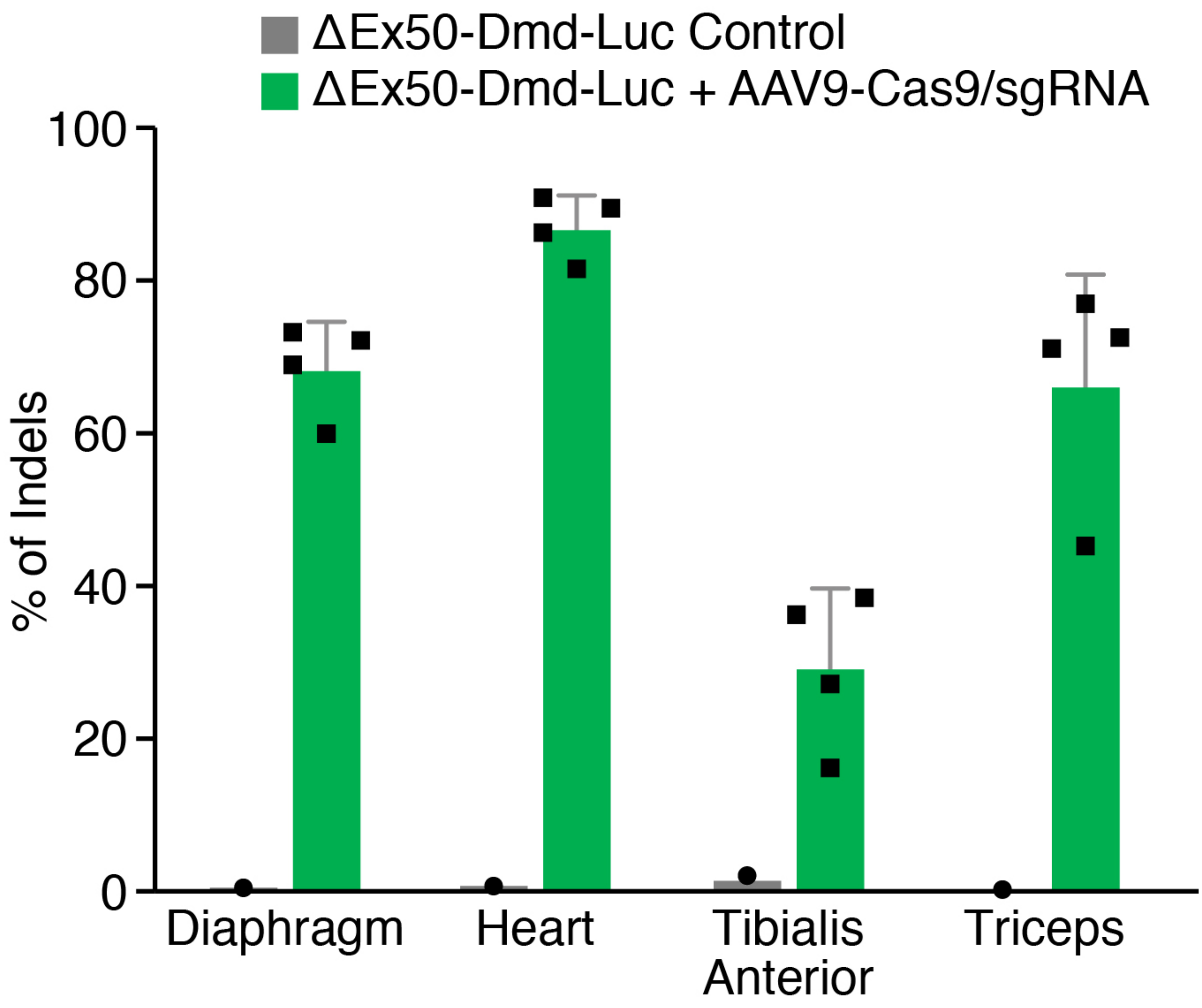

Supplementary Figure 11 In vivo *Dmd* gene editing after systemic delivery of AAV9-Cas9 and AAV9-sgRNA-51. Percentage of indels detected at exon 51 after AAV9-Cas9 and AAV9-sgRNA-51 treatment using tracking indels by decomposition (TIDE) analysis of RT-PCR products from diaphragm, heart, tibialis anterior and triceps muscle samples of untreated ΔEx50-Dmd-Luc mice and ΔEx50-Dmd-Luc mice injected with AAV9-Cas9 and AAV9-sgRNA-51. n=4.

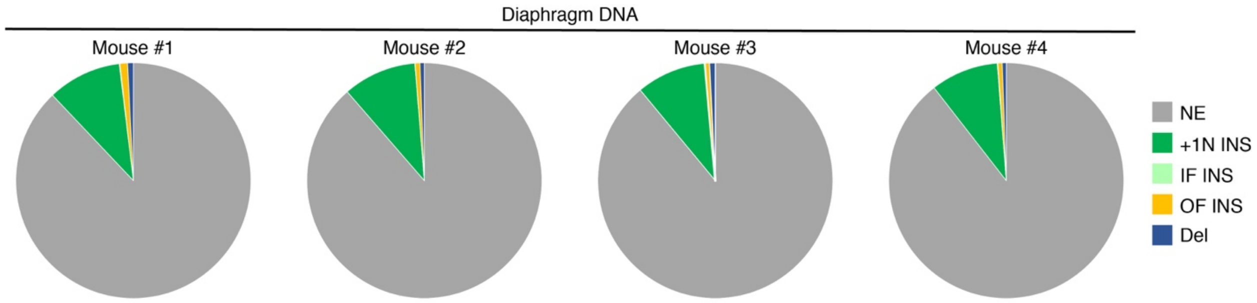

Type	% reads	reads
NE	87.74	120195
+1N INS	10.14	13887
IF INS	0.10	134
OF INS	1.04	1423
Del	0.78	1354

Type	% reads	reads
NE	88.41	108050
+1N INS	10.07	12313
IF INS	0.05	59
OF INS	0.65	800
Del	0.59	990

Type	% reads	reads
NE	88.51	107426
+1N INS	9.56	11607
IF INS	0.07	91
OF INS	0.54	652
Del 01-10	0.80	1595

Type	% reads	reads
NE	89.27	180278
+1N INS	9.28	18749
IF INS	0.11	217
OF INS	0.58	1167
Del 01-10	0.54	1531

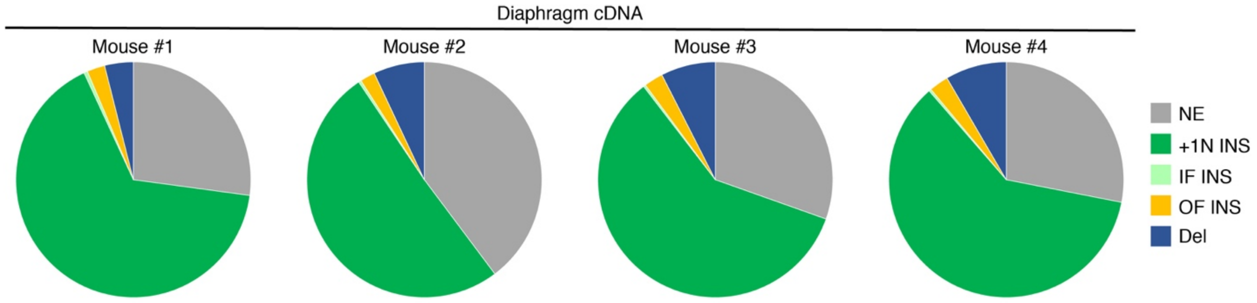

Type	% reads	reads
NE	26.03	34270
+1N INS	63.29	83309
IF INS	0.48	635
OF INS	2.35	3100
Del	3.78	10324

Type	% reads	reads
NE	39.75	57886
+1N INS	50.82	73997
IF INS	0.36	520
OF INS	2.06	3000
Del	7.01	10212

Type	% reads	reads
NE	30.42	41306
+1N INS	59.13	80292
IF INS	0.32	428
OF INS	2.63	3570
Del	7.51	10197

Type	% reads	reads
NE	28.08	19036
+1N INS	60.52	41032
IF INS	0.32	217
OF INS	2.67	1807
Del	8.42	5706

Supplementary Figure 12 In vivo *Dmd* gene editing 10 weeks after systemic delivery of AAV9-Cas9 and AAV9-sgRNA-51. Genomic deep sequencing analysis of DNA from diaphragm muscles of ΔEx50-Dmd-Luc mice injected with AAV9-Cas9 and AAV9-sgRNA-51 10 weeks after systemic injection (top panel). Genomic PCR amplicons generated across the exon 51 target site were analyzed by deep sequencing. RT-PCR (cDNA) deep sequencing analysis of RNA from diaphragm muscles of ΔEx50-Dmd-Luc mice injected with AAV9-Cas9 and AAV9-sgRNA-51 10 weeks after systemic injection

(lower panel). RT-PCR with primers for sequences in exons 48 and 53 were subjected to amplicon deep-sequencing. The alignment of reads at the cleavage site was further analyzed and regrouped in non-edited (NE), single nucleotide insertion (+1N INS), in frame insertion (IF INS), out of frame insertion (OF INS) and deletion (DEL) groups. n=4.

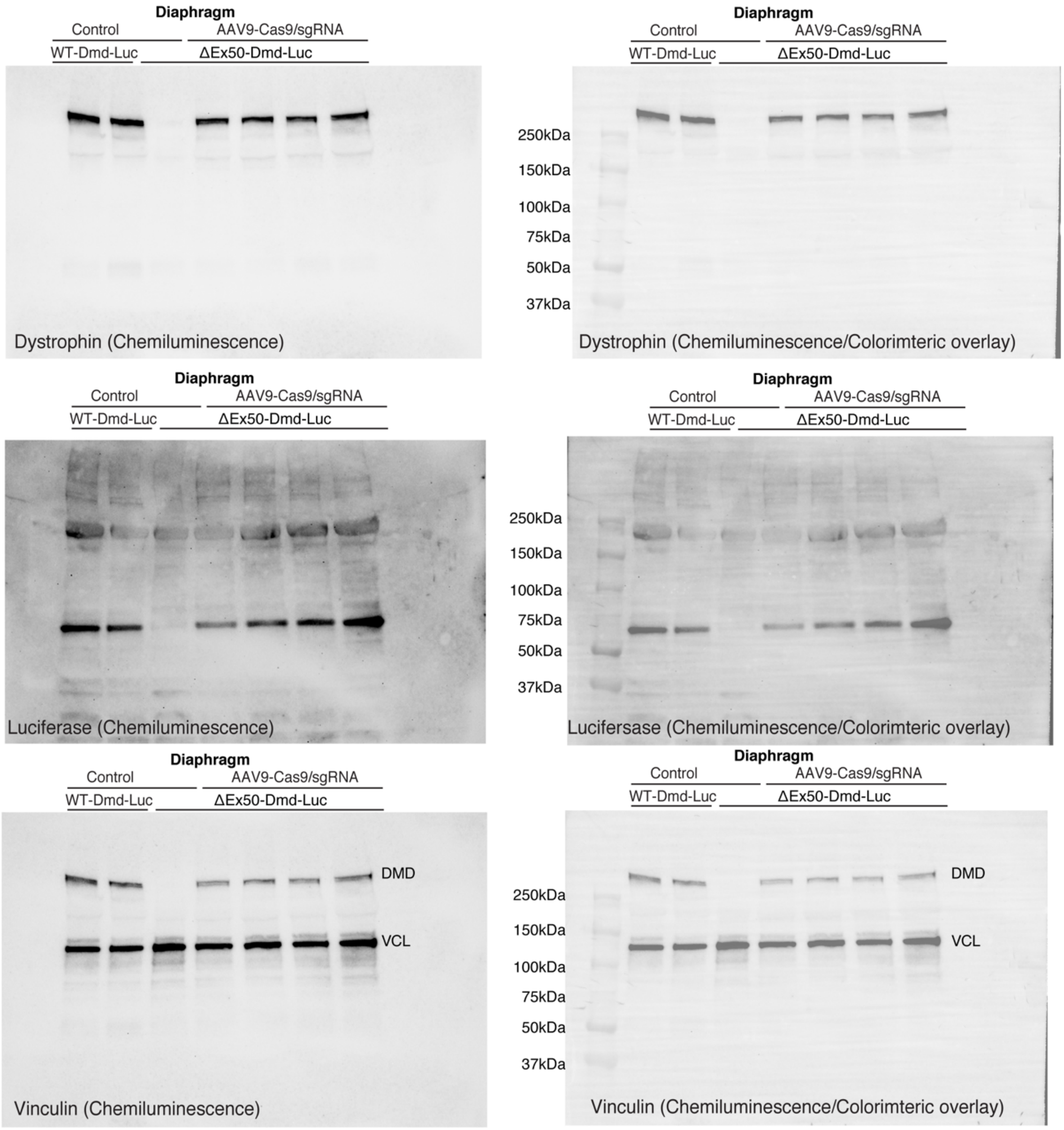

Supplementary Figure 13 Full diaphragm western blot scan corresponding to Figure 4.

Dystrophin (DMD) and vinculin (VCL).

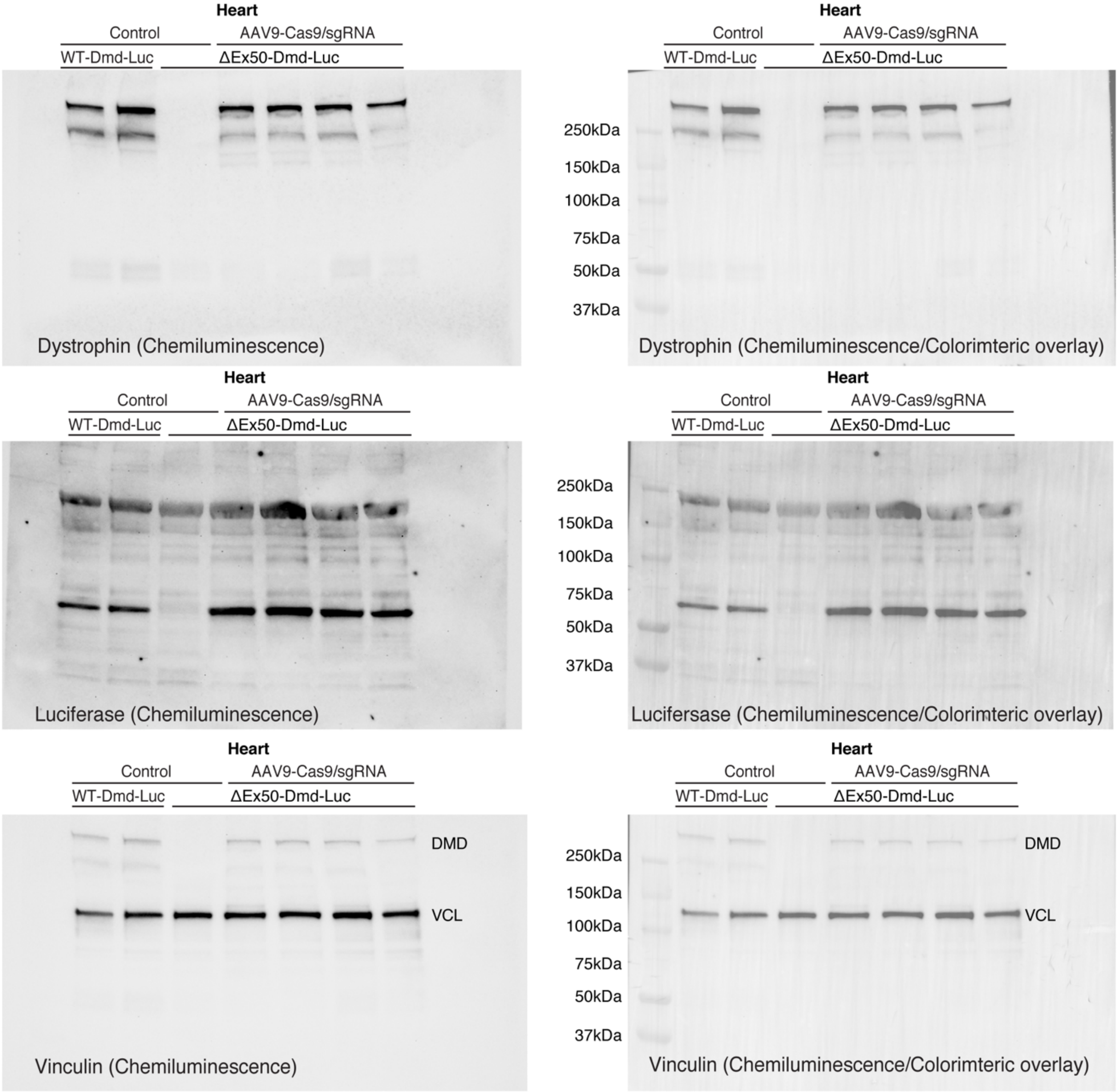

Supplementary Figure 14 Full heart western blot scan corresponding to Figure 4.

Dystrophin (DMD) and vinculin (VCL).

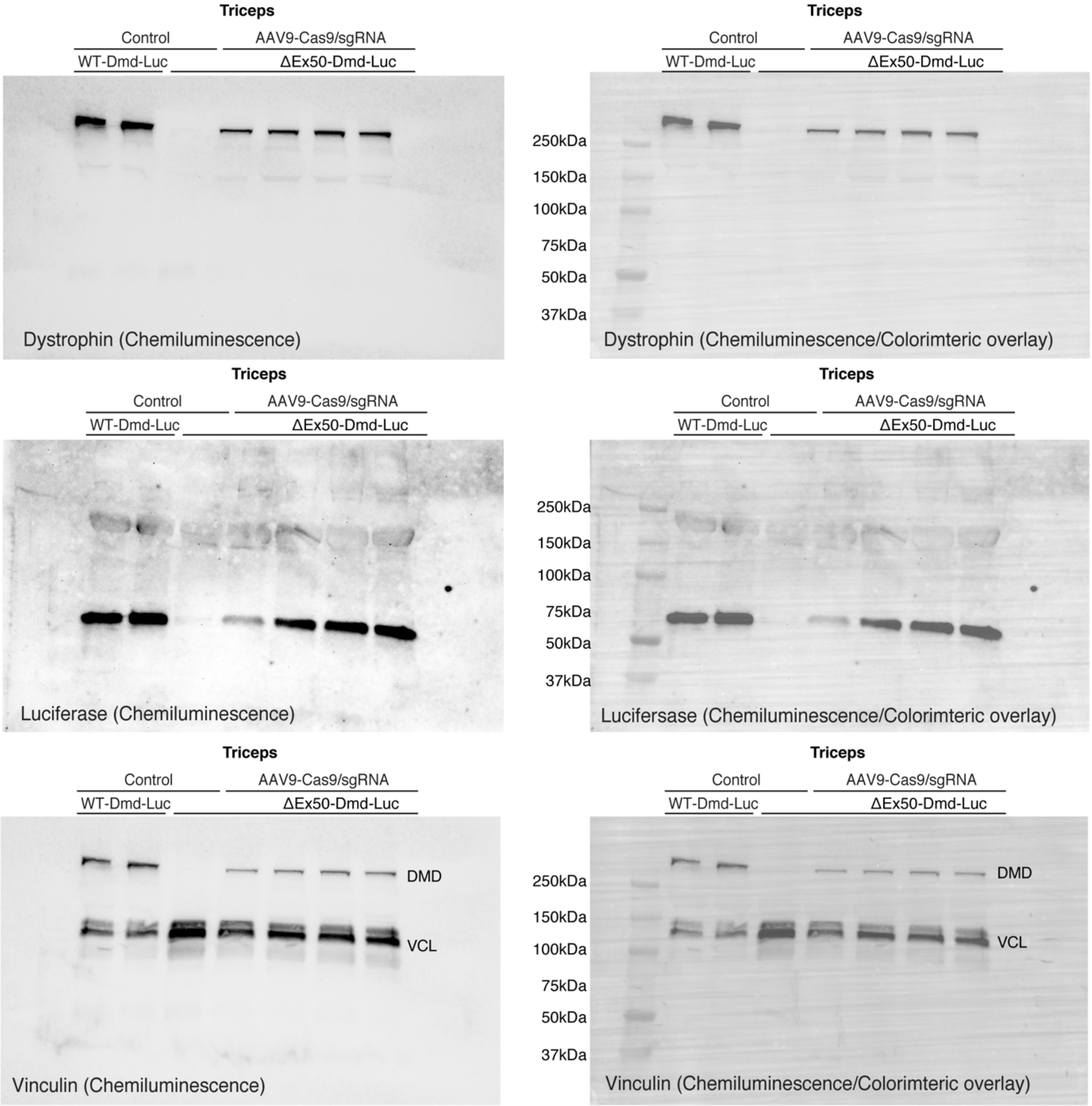

Supplementary Figure 15 Full triceps western blot scan corresponding to Figure 4.

Dystrophin (DMD) and vinculin (VCL).

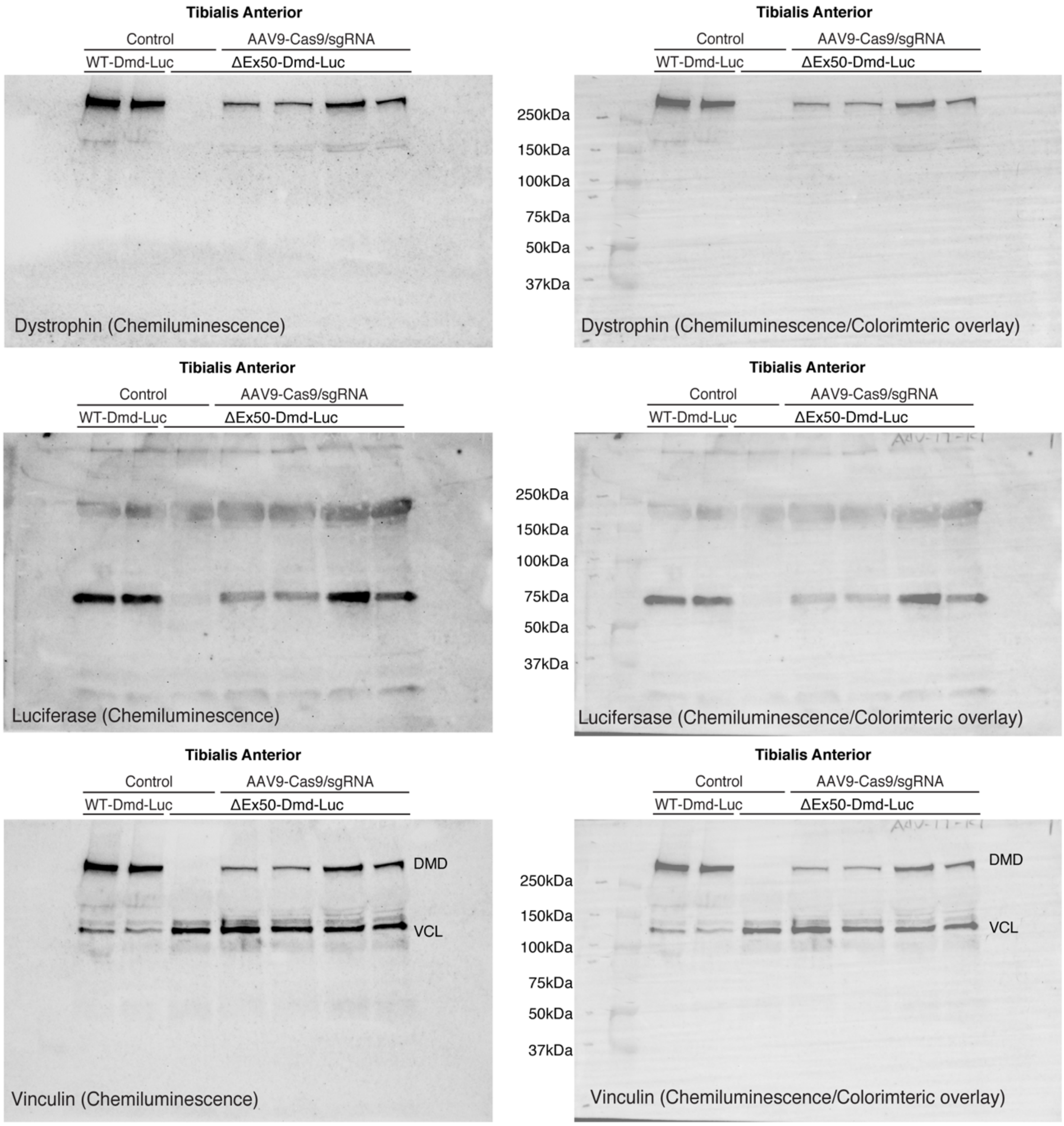

Supplementary Figure 16 Full tibialis anterior western blot scan corresponding to Figure 4. Dystrophin (DMD) and vinculin (VCL).

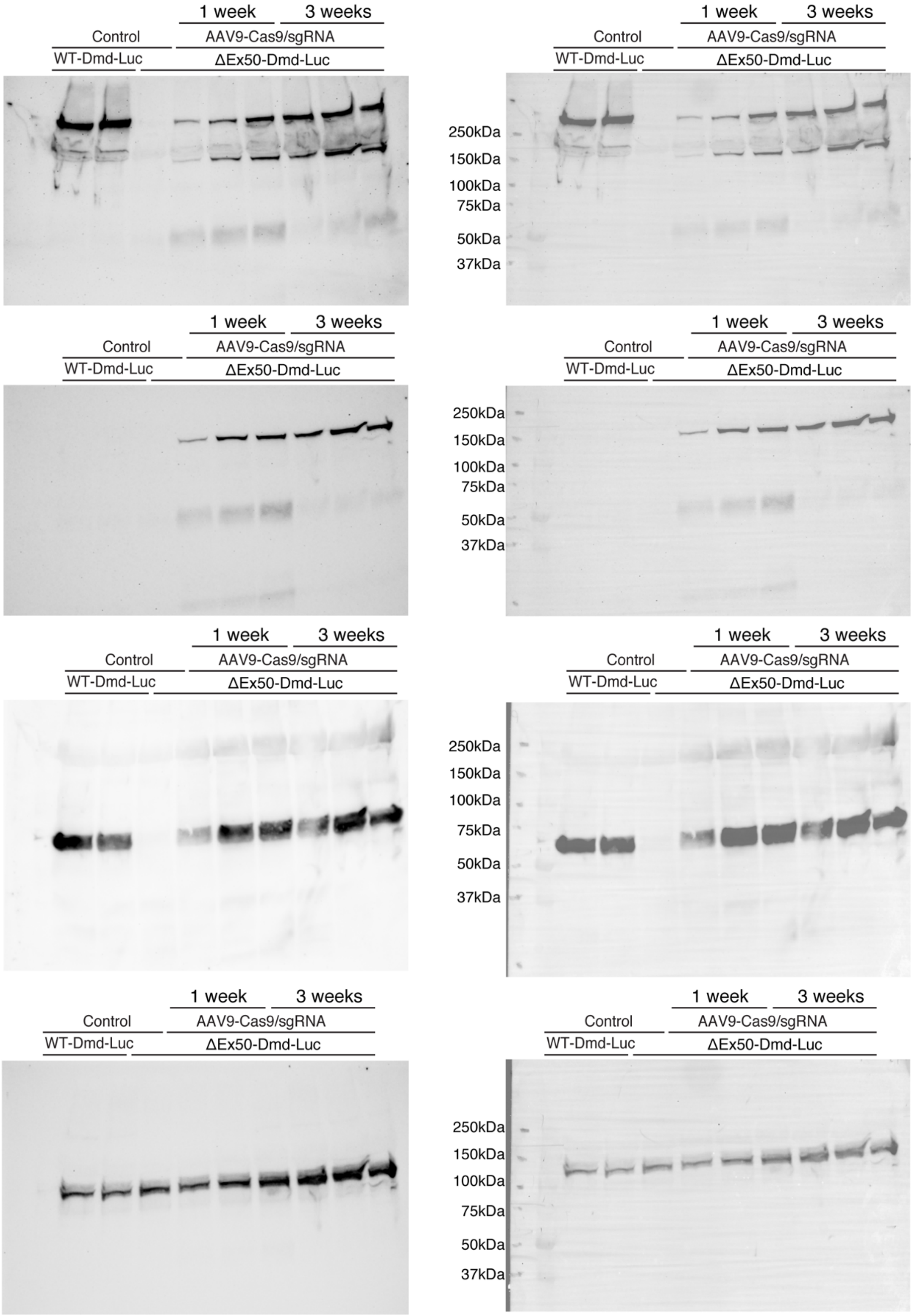

Supplementary Figure 17 Full tibialis anterior western blot scan corresponding to Supplementary Figure 6.

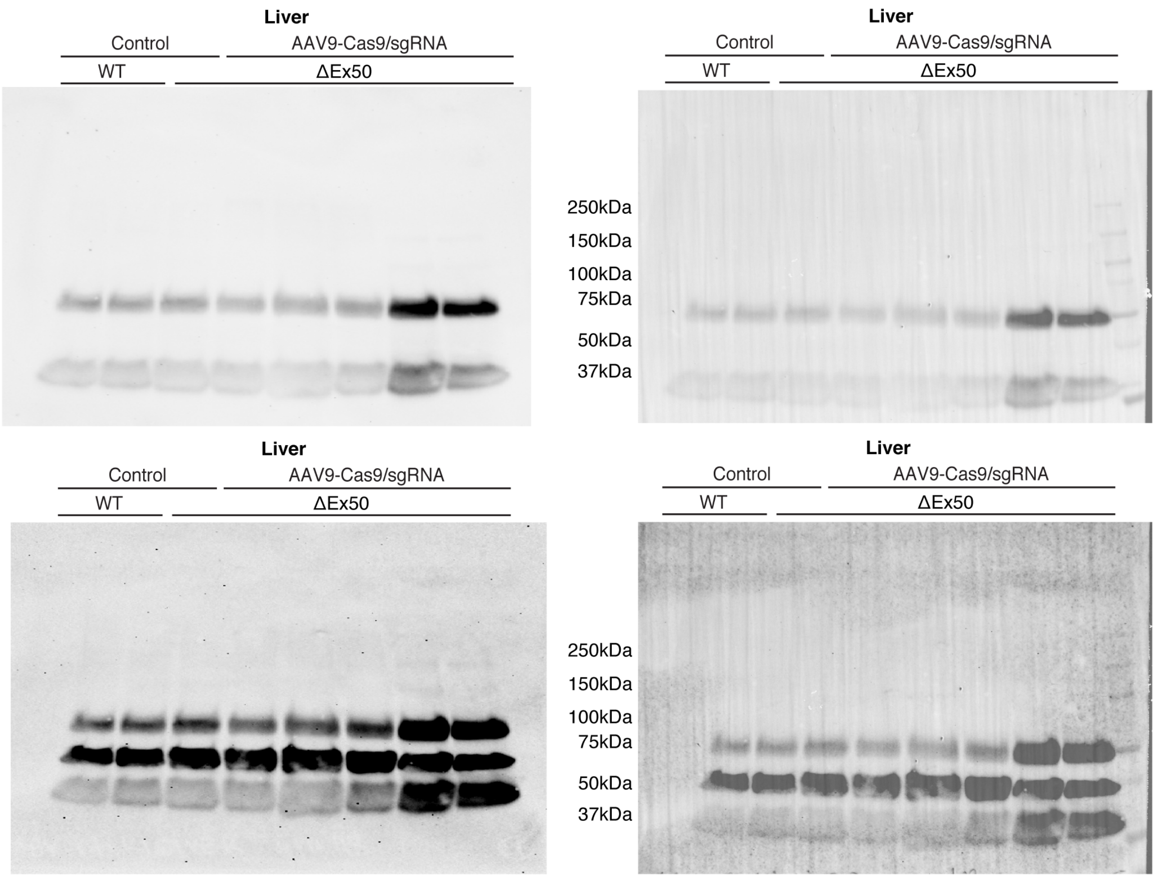

Supplementary Figure 18 Full liver western blot scan corresponding to Supplementary Figure 10.

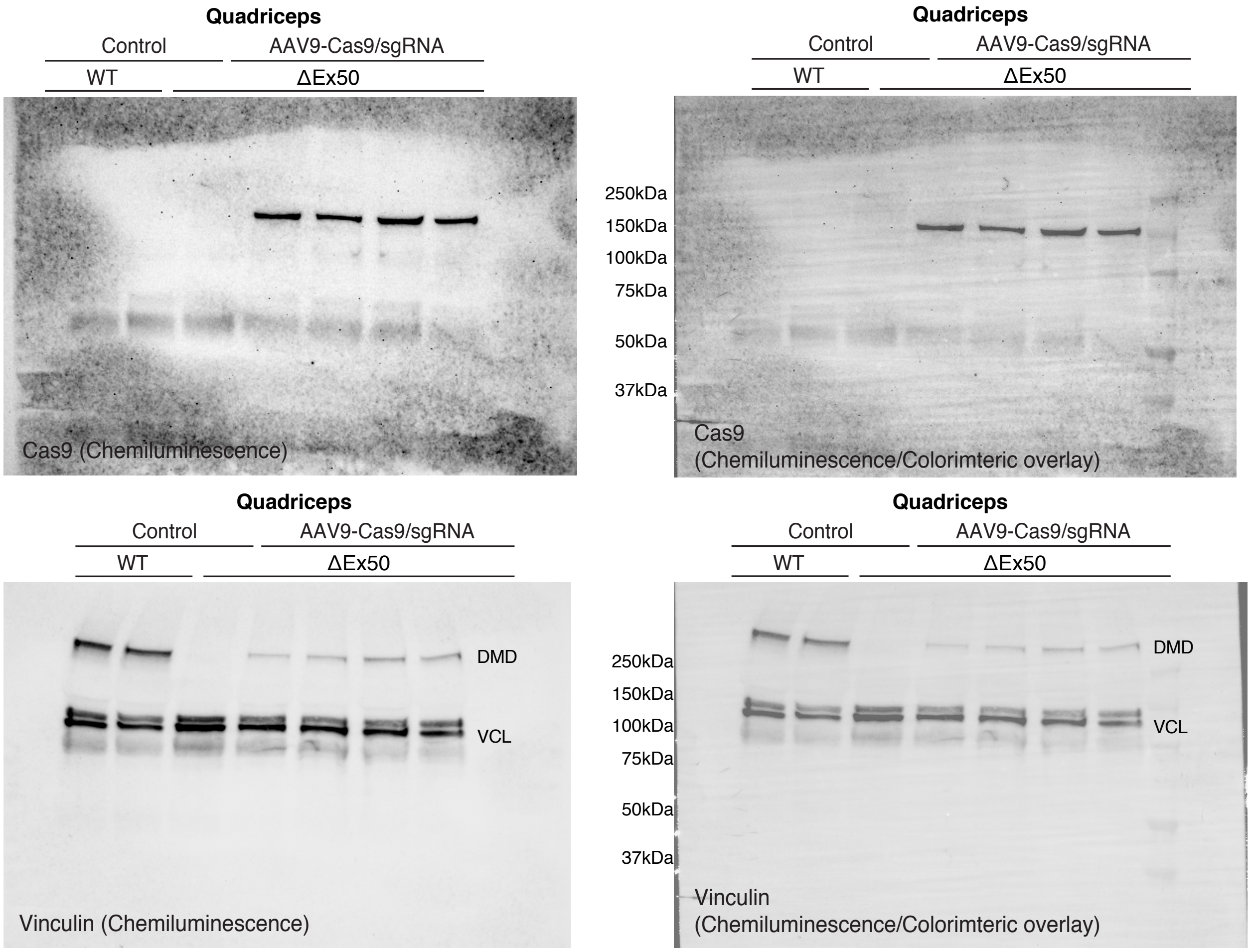

Supplementary Figure 19 Full quadriceps western blot scan corresponding to Supplementary Figure 10. Dystrophin (DMD) and vinculin (VCL).

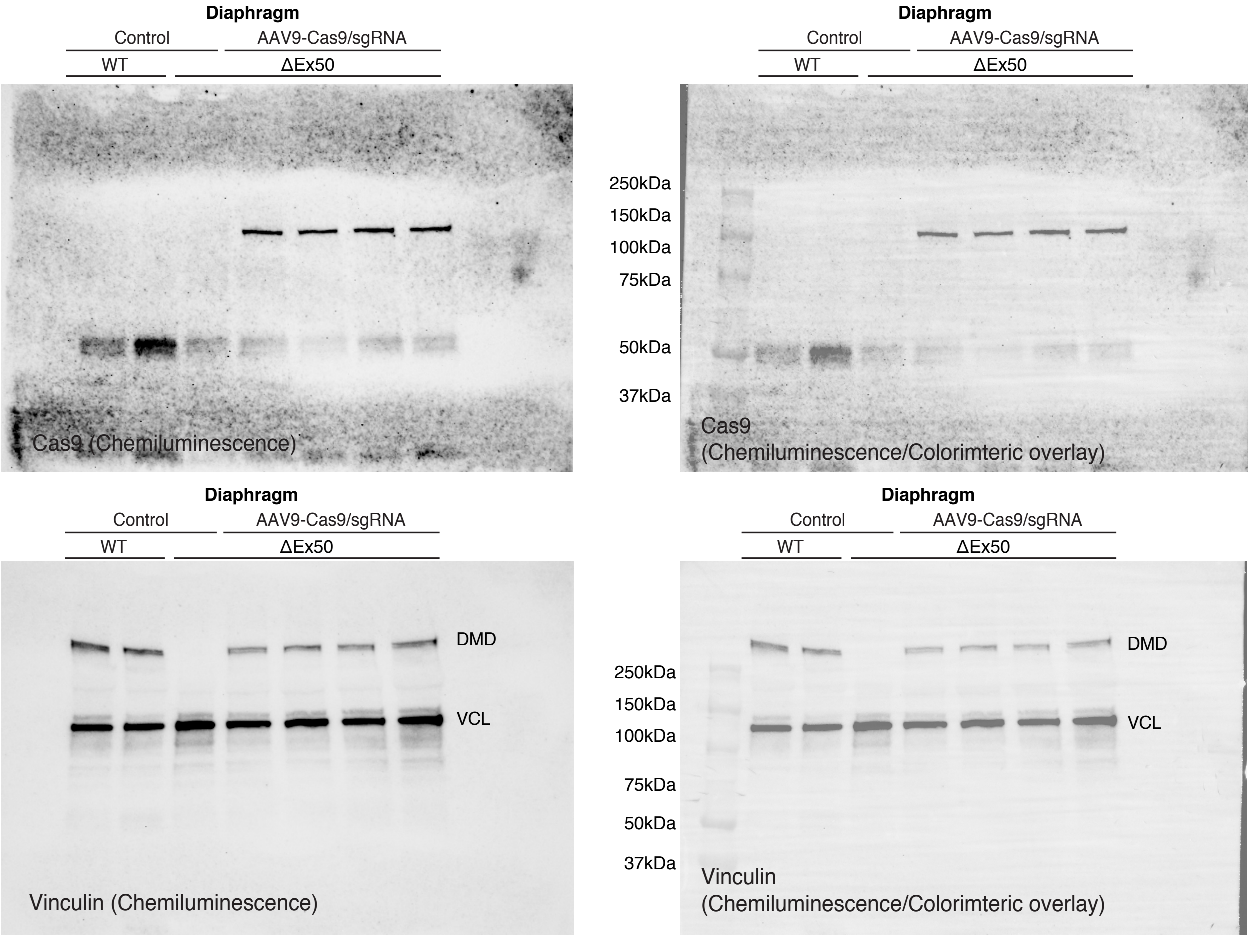

Supplementary Figure 20 Full diaphragm western blot scan corresponding to Supplementary Figure 10. Dystrophin (DMD) and vinculin (VCL).

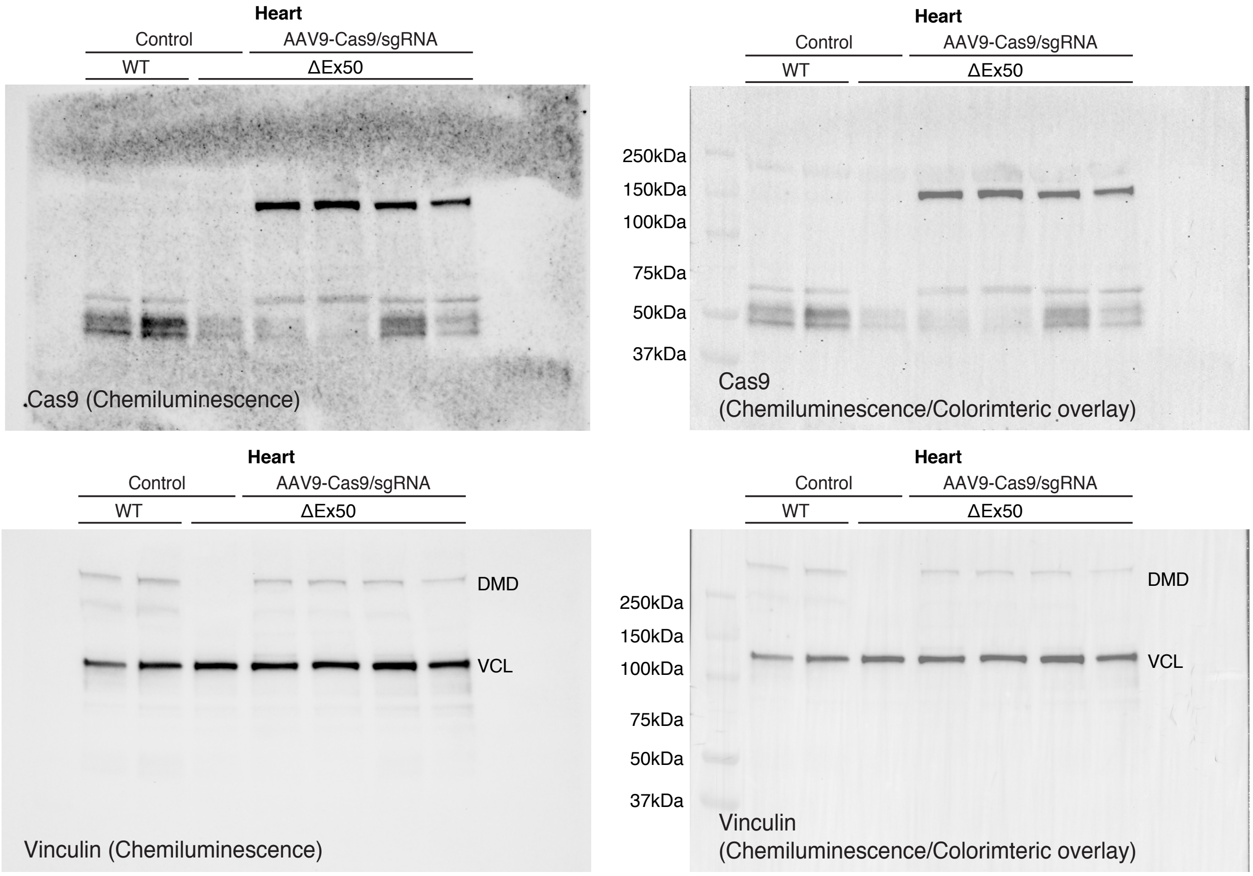

Supplementary Figure 21 Full heart western blot scan corresponding to Supplementary Figure 10. Dystrophin (DMD) and vinculin (VCL).

Supplementary Table 1 Primer sequences.

Experiment	Primer name	Primer sequence (5'-3')
sgRNA for Targeting the end exon 79 for KI	*Dmd*-Ex79-Top	CACCGAGGCTTCCTACATTGTGTCC
	Dmd-Ex79-Bottom	AAACGGACACAATGTAGGAAGCCTC
sgRNA for exon 50	*Dmd* exon 50-Top-1	CACCGAATGATGAGTGAAGTTATAT
	Dmd exon 50-Bottom-1	AAACATATAACTTCACTCATCATTC
	Dmd exon 50-Top-2	CACCGGTTTGTTCAAAAGCGTGGCT
	Dmd exon 50-Bottom-2	AAACAGCCACGCTTTTGAACAAAC
Genotyping of ΔEx50	Geno-Ex50-F	GGATTGACTGAAATGATGGCCAAGG
	Geno-Ex-50-R	CTGCCACGATTACTCTGCTTCCAG
Genotyping of KI Luciferase	Geno-KLuci-F	AGCAGGCAGAGAAGGTGGTA
	Geno-Kluci-WT-R	GCGTGTGTGTTTGTTTAGGG
	Geno-Kluci-KI-R	GGGCGTATCTCTTCATAGCCTT
sgRNA for exon 51	*Dmd*-Ex51-Top	CACCG CAC TAG AGT AAC AGT CTG AC
	Dmd-Ex51-Bottom	AAAC GTCAGACTGTTACTCTAGTG C
RT-PCR primer flanking exon 48 and exon 53 TIDE	*Dmd*-Ex48-F	AGGTTCACTTAAAAGATTTTAGGCAG
	Dmd-Ex52-R	TAACATTTCATTCAACTGTTGTCTCC
Primer flanking genomic exon 51 locus TIDE	*Dmd*-Ex48-F	GATAAATCCTGAAAATTCCCCAATA
	Dmd-Ex52-R	TTTCACCCTAACTTTTCATCCCTT
Primers for Mi-seq mouse genomic DNA amplicon deep sequencing	*Dmd*-Ex51-Mi-seq-F	TCGTCGGCAGCGTCAGATGTGTATAAGAGACAGGAAATTTTACCTCAAACTGTTGCTTC
	Dmd-Ex51-Mi-seq-F	GTCTCGTGGGCTCGGAGATGTGTATAAGAGACAGGAGGGAAATGGAAAGTGACAATATAC
Primers for Mi-seq Mouse cDNA amplicon deep sequencing	*Dmd*-Ex51-cDNA-Mi-seq-F	TCGTCGGCAGCGTCAGATGTGTATAAGAGACAGGGAAATTTATAACCAACCAAGTCAGG
	Dmd-Ex51-cDNA-Mi-seq-R	GTCTCGTGGGCTCGGAGATGTGTATAAGAGACAGAATGATTGTTCTAGCTTCTTGATTGC
Primers for Mi-Seq for PCR with Bar Codes	Univ-F	AATGATACGGCGACCACCGAGATCTACACTCGTCGGCAGCGTC
	BC1-F	CAAGCAGAAGACGGCATACGAGAT**ACATCG**GTCTCGTGGGCTCGG
	BC2-F	CAAGCAGAAGACGGCATACGAGAT**TGGTCA**GTCTCGTGGGCTCGG
	BC3-F	CAAGCAGAAGACGGCATACGAGAT**CACTGT**GTCTCGTGGGCTCGG
	BC4-F	CAAGCAGAAGACGGCATACGAGAT**ATTGGC**GTCTCGTGGGCTCGG
	BC5-F	CAAGCAGAAGACGGCATACGAGAT**GATCTG**GTCTCGTGGGCTCGG
	BC6-F	CAAGCAGAAGACGGCATACGAGAT**TACAAG**GTCTCGTGGGCTCGG
	BC7-F	CAAGCAGAAGACGGCATACGAGAT**CGTGAT**GTCTCGTGGGCTCGG
	BC8-F	CAAGCAGAAGACGGCATACGAGAT**GCCTAA**GTCTCGTGGGCTCGG
	BC9-F	CAAGCAGAAGACGGCATACGAGAT**TCAAGT**GTCTCGTGGGCTCGG
	BC10-F	CAAGCAGAAGACGGCATACGAGAT**AGCTAG**GTCTCGTGGGCTCGG
	BC11-F	CAAGCAGAAGACGGCATACGAGAT**GTCGTC**GTCTCGTGGGCTCGG

	BC12-F	CAAGCAGAAGACGGCATACGAGAT**CGATTA**GTCTCGTGGGCTCGG
	BC13-F	CAAGCAGAAGACGGCATACGAGAT**GAATGA**GTCTCGTGGGCTCGG
	BC14-F	CAAGCAGAAGACGGCATACGAGAT**CTTCGA**GTCTCGTGGGCTCGG
	BC15-F	CAAGCAGAAGACGGCATACGAGAT**CTCTAC**GTCTCGTGGGCTCGG
	BC16-F	CAAGCAGAAGACGGCATACGAGAT**AGGAAT**GTCTCGTGGGCTCGG
	BC17-F	CAAGCAGAAGACGGCATACGAGAT**GCTACC**GTCTCGTGGGCTCGG
	BC18-F	CAAGCAGAAGACGGCATACGAGAT**ATCAGT**GTCTCGTGGGCTCGG
	BC19-F	CAAGCAGAAGACGGCATACGAGAT**TATACT**GTCTCGTGGGCTCGG
	BC20-F	CAAGCAGAAGACGGCATACGAGAT**CAACAA**GTCTCGTGGGCTCGG
	BC21-F	CAAGCAGAAGACGGCATACGAGAT**GTTGTT**GTCTCGTGGGCTCGG
	BC22-F	CAAGCAGAAGACGGCATACGAGAT**TCGGTT**GTCTCGTGGGCTCGG
	BC23-F	CAAGCAGAAGACGGCATACGAGAT**AGTATT**GTCTCGTGGGCTCGG
	BC24-F	CAAGCAGAAGACGGCATACGAGAT**TTAATT**GTCTCGTGGGCTCGG
	BC25-F	CAAGCAGAAGACGGCATACGAGAT**GAGTGT**GTCTCGTGGGCTCGG
	BC26-F	CAAGCAGAAGACGGCATACGAGAT**AATGGT**GTCTCGTGGGCTCGG
	BC27-F	CAAGCAGAAGACGGCATACGAGAT**GGCGGT**GTCTCGTGGGCTCGG
